# Chain Conformation and Crosslink Density as Independent Mechanistic Levers in Comb-Brush Particle Artificial Solid Electrolyte Interfaces for Lithium–Metal Batteries

**DOI:** 10.1021/acsapm.6c02177

**Published:** 2026-08-19

**Authors:** Verena Kempkes, Hanshu Wu, Jirameth Tarnsangpradit, Elizabeth E. DiFiglia, Bailey Williams, Kwangmo Go, Michael R. Bockstaller, Jay F. Whitacre, Krzysztof Matyjaszewski

**Affiliations:** † Department of Chemistry, 6612 Carnegie Mellon University, 4400 Fifth Avenue, Pittsburgh, Pennsylvania 15213, United States; ‡ Department of Materials Science and Engineering, Carnegie Mellon University, 5000 Forbes Avenue, Pittsburgh, Pennsylvania 15213, United States

**Keywords:** Lithium−metal batteries, artificial solid electrolyte interface, comb-brush particles, entanglements, crosslinking

## Abstract

Artificial solid electrolyte interfaces (aSEIs) based on organic–inorganic hybrids offer a promising strategy to stabilize lithium–metal anodes by combining mechanical strength and ionic conductivity. Herein, entanglement- and crosslink-enabled comb-brush particles (CBPs) were synthesized by grafting oligo­(ethylene oxide) methyl ether methacrylate (OEOMA_500_) and 2-(dimethylamino)­ethyl methacrylate (DMAEMA) from silica nanoparticles via atom transfer radical polymerization. Systematic variation of DMAEMA content enabled control over polymer conformation and mechanical properties. Increasing DMAEMA incorporation reduced polymer extension and enhanced chain entanglement, resulting in higher storage moduli and decreased creep deformation. Blended with LiTFSI, higher DMAEMA contents led to reduced ionic conductivity. Despite this trade-off, CBPs containing 5 – 25 wt % DMAEMA demonstrated stable symmetric cycling over 2000 h with low overpotentials and uniform lithium deposition. Higher DMAEMA fractions, on the other hand, resulted in soft shorting and dendrite formation. Topical crosslinking via quaternization further tuned interphase stability. Bromide-based crosslinkers provided improved cycling stability compared to their triflate analogs. Long-chain Br-PEO_2k_-Br enabled interparticle crosslinking and overpotentials as low as 40 mV. These results demonstrate that balancing entanglements, ionic conductivity, and controlled crosslinking density is critical for designing robust hybrid aSEIs for lithium–metal batteries.

## Graphical abstract

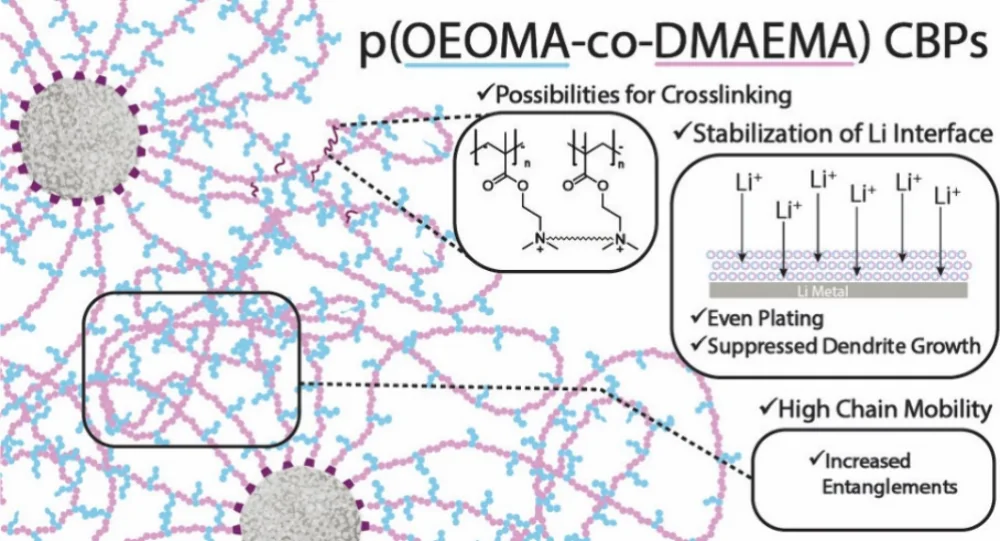

## 1 Introduction

Due to the demand for high-energy density rechargeable batteries, lithium–metal, with its high theoretical specific capacity of 3860 mAh g^–1^, has emerged as one of the most promising anode materials. With the additional advantages of the lowest electrochemical potential among metals (−3.04 V vs SHE) and low density of 0.534 g cm^–3^, the lithium–metal anode is widely regarded as the ‘holy grail’ for energy storage systems.^1–6^ Yet, despite this promise, potential applications ranging from electric vehicles to grid-scale storage are hampered by severe challenges associated with interfacial instability.^7–9^

The root cause of these challenges lies in the high chemical reactivity and infinite relative volume change of lithium metal during plating and stripping. Upon initial contact with liquid or solid electrolytes, lithium spontaneously forms a passivation layer known as the solid electrolyte interphase (SEI).^10^ Ideally, the SEI should be ionically conductive, electronically insulating, mechanically robust, and chemically stable. However, in conventional systems, the native SEI is heterogeneous, mechanically fragile, and dynamically unstable.^11^ Repeated lithium deposition and dissolution induce stress accumulation and fracture within the SEI, leading to nonuniform lithium flux, localized current hotspots, and ultimately dendrite and ‘dead’ lithium formation. These dendritic structures not only decrease Coulombic efficiency but also pose severe safety hazards due to potential internal short-circuiting.^12,13^ Consequently, significant efforts have focused on engineering protective interfacial layers that can stabilize lithium–metal surfaces during cycling.

Among these approaches, artificial solid electrolyte interfaces (SEIs) have emerged as an effective strategy to regulate lithium-ion deposition, suppress dendritic growth, and stabilize the interface between electrode and electrolyte.^14^ Inorganic as well as organic materials have been used for these protective layers.^15,16^ Inorganic aSEIs are typically composed of lithium-containing compounds such as LiF, Li_2_O, Li_3_N, Li_3_PO_4_, lithium halides or sulfide- and oxide-based ceramics. Due to their characteristic high mechanical strength, chemical stability and intrinsic lithium-ion conductivity, these inorganic aSEIs have been utilized in lithium–metal batteries.^17,18^ However, most inorganic SEIs remain limited due to their structural brittleness as well as poor adaptability to volume changes.^19^

To overcome these limitations. organic SEIs have attracted increasing attention due to their high synthetic versatility and mechanical compliance. In particular, polymer-based aSEIs have attracted significant attention due to their chemical tunability, conformal coating capability, and intrinsic flexibility.^20–22^ Importantly, tailoring polymer architecture enables simultaneous optimization of ionic conductivity and mechanical strength which are critically interdependent in lithium–metal systems.

Among polymer architectures, comb-brush structures represent a uniquely versatile platform for designing functional interphases.^23–27^ Comb-brush polymers consist of a backbone with densely grafted side chains, allowing independent control over segmental mobility, ion solvation, and mechanical reinforcement. Incorporation of poly­(ethylene oxide) (PEO)-like side chains facilitates lithium-ion transport through segmental motion and coordination with ether oxygen atoms.^28^ Meanwhile, the backbone can be engineered to provide structural integrity and compatibility with the lithium surface. This architectural decoupling of mechanical and transport properties enables the rational design of materials that overcome the classical trade-off between ionic conductivity and mechanical strength in polymer electrolytes.

Beyond tuning molecular architecture alone, grafting polymer chains onto nanoscale substrates provides an additional dimension of structural and functional control. In recent years, comb-brush particles (CBPs) have emerged as promising components for functional composite materials by decoupling mechanical and transport functions onto distinct structural elements.^29–31^ When densely grafted with polymer brushes, nanoparticles behave as soft colloidal objects with tunable interparticle interactions. Such hybrid systems offer an opportunity to construct artificial interphases that are simultaneously robust, ionically conductive, and resistant to crack propagation.^32^

A critical but underexplored factor governing the performance of these brush-based systems is polymer entanglement, which strongly influences the mechanical and viscoelastic behavior of polymer materials. In brush-based materials, due to the effect of steric constraints, high molecular masses are required to promote physical entanglements.^33^ These entanglements reduce flow under mechanical stress without requiring permanent chemical bonding.^34,35^ During the cycling process in lithium–metal batteries, entangled polymer networks can accommodate interfacial volume changes while maintaining the required contact with the electrode surface. However, an inherent competition between entanglement and ionic conductivity exists. While entanglements enhance mechanical robustness, it can also restrict segmental motion and thus hinder ion transport. Importantly, entanglement-driven reinforcements preserve a degree of chain mobility necessary for ion conduction, offering an advantage over highly crosslinked systems in which excessive network rigidity more severely compromises ionic conductivity.^36,37^

However, physical entanglements possess a finite lifetime and can disengage under prolonged stress. Hence, they permit creep and time-dependent plastic deformation, which may lead to nonuniform swelling and spatially heterogeneous mechanical strength throughout the aSEI layer. To further stabilize the interphase, the introduction of chemical crosslinks provides a complementary reinforcement mechanism.^38–40^ The combination of entanglement and crosslinking offers a synergistic approach to interphase design. Entanglements provide dynamic adaptability and toughness, while crosslinks deliver enhanced mechanical reinforcement and stability. Together, these features enable the construction of aSEIs capable of withstanding the substantial volume changes of lithium metal during repeated plating and stripping. Moreover, the presence of ion-solvating side chains ensures that lithium-ion transport pathways remain continuous throughout the interphase, promoting homogeneous current distribution and suppressing dendrite nucleation. Recent studies have demonstrated that mechanically robust interphases with moduli exceeding a critical threshold can effectively suppress dendritic growth by homogenizing lithium-ion transport.^41–43^

In this work, entanglements and crosslinking within CBP-based aSEIs were systematically investigated to further understand the structure–property relationships crucial to their performance in lithium–metal batteries. By tailoring macromolecular architecture to incorporate less bulky side chains, CBPs achieved effective interparticle entanglement that strengthened film cohesion, improved interfacial elasticity, and enhanced ion transport pathways. The incorporation of 2-(dimethylamino)­ethyl methacrylate (DMAEMA) introduced amino functionalities, which have been shown to stabilize the lithium interface.^44–46^ Additionally, these amino groups enabled crosslinking via quaternization, providing an additional strategy to tube the mechanical and electrochemical properties of the aSEIs. Through the implementation of entanglement-enabled brush architectures and chemical crosslinking, this work establishes a new molecular design strategy for polymer–particle interphases capable of simultaneously balancing ionic conductivity, mechanical integrity, and long-term electrochemical stability. Beyond advancing artificial SEI design, the findings provide fundamental insights into how macromolecular architecture and interfacial physics can be leveraged to stabilize reactive metal anodes and enable the next generation of high-energy batteries.

## 2 Results and Discussion

### 2.1 CBP Synthesis

The mechanistic premise of this work is that brush chain conformation determines the degree of entanglement and the density of the ion-solvating segments which affect the mechanical and transport properties of the aSEI simultaneously. To test this, comb-brush particles (CBPs) with varying incorporation of bulky and compact monomers were synthesized. 3-(Chlorodimethylsilyl)­propyl α-bromoisobutyrate was immobilized on silica nanoparticles (average diameter = 15.8 nm) to functionalize the surface.^47^ CBPs were synthesized by polymerizing oligo­(ethylene oxide) methyl ether methacrylate (average molecular mass = 500 g mol^–1^, OEOMA_500_) and 2-(dimethylamino)­ethyl methacrylate (DMAEMA) from the functionalized silica nanoparticles via activators regenerated by electron transfer atom transfer radical polymerization (ARGET ATRP).^48,49^ As previously reported by our group, polymer chains grafted from nanoparticles using purely OEOMA_500_ as the monomer – such as N_0_ – exhibited a highly extended conformation, corresponding to a scaling factor of 0.9.^33^ To investigate the effect of incorporating a less bulky monomer, DMAEMA was incorporated at monomer ratios ranging from 15 to 65 mol% relative to OEOMA and samples were named N_DMAEMAwt %_ (Table 1).

**Table 1:** Molecular Properties and Scaling Factors of SiO_2_-g-(DMAEMA-co-OEOMA_500_) Samples

Entry	DMAEMA/OEOMA_ini_ [wt%]	DMAEMA/OEOMA_ini_ [mol %]	*M*_n,app_ ^a^ [kDa]	*Đ* []	*f*_inorg_ ^b^ [%]	GD^c^ [nm^–2^]	DMAEMA^d^ mol %	Scaling factor^e^
N_0_ ^33^	0/100	0/100	52.2	1.42	8.42	0.72	0	0.9
N_5_	5/95	15/85	83.7	1.45	5.37	0.73	18.2	-
N_10_	10/90	28/72	73.2	1.41	7.30	0.61	30.0	-
N_20_	20/80	48/52	65.3	1.40	7.23	0.69	52.3	0.7
N_25_	25/75	55/45	72.6	1.45	5.88	0.76	58.4	0.7
N_30_	30/70	60/40	54.8	1.35	6.31	0.95	63.8	0.6
N_35_	35/65	65/35	59.2	1.36	6.47	0.85	70.3	0.4

a Determined via gel permeation chromatography in DMF at 50 °C, calibrated using polymethylmethacrylate standards.

b Determined via thermogravimetric analysis.

c Calculated by eq 1.

d Determined via ^1^H NMR.

e Determined by plotting the brush height against the molecular mass.

Molar masses in the range of 52 to 83 kDa were obtained while maintaining control over the polymerization, with dispersities of approximately 1.40. Inorganic fractions (*f*_inorg_) were kept low at approximately 5 – 8% to ensure sufficient amount of polymer chains to facilitate lithium-ion transportation. Grafting densities (GDs) were calculated using eq 1 in which *f*_inorg_ is the inorganic fraction, *N*_A_ is the Avogadro constant, ρ is the density of the inorganic core, d is the diameter of the core and *M*_n_ is the number-average molecular mass of the grafted polymer chains:

1 $$\mathrm{GD}=\frac{\left(1-f_{\mathrm{inorg}}\right)N_{A}\rho d}{6f_{\mathrm{inorg}}M_{n}}$$

GD values revealed the steric mechanism directly. While GDs for N_0_ – N_20_ with low DMAEMA contents exhibited values of 0.6 – 0.7 nm^–2^, higher DMAEMA incorporations in N_25_ – N_35_ resulted in an increase to 0.8 – 0.9 nm^–2^. Due to the higher amount of the bulky monomer in N_0_ – N_20_, the polymer chains occupy a larger volume. Consequently, initiators in proximity to already propagated initiators are no longer available for polymerization, leading to the reduced GDs. As DMAEMA exceeded 50 mol % in N_25_ – N_35_, its smaller side group relieved the steric crowding, allowing for more initiators to participate in propagation.

The incorporation of DMAEMA was verified and quantified by ^1^H NMR. All CBP samples exhibited slightly higher incorporation ratios compared to the initial monomer ratios, with an average increase of ∼ 3 mol %. This observation was ascribed to near-equal incorporation of both monomers during the polymerization, with a marginal kinetic preference toward the incorporation of DMAEMA.

To determine the polymer conformation, additional samples were synthesized for each polymer composition with DMAEMA incorporation above 10 wt % and scaling factor analysis was performed (Table S1).^33,50^ As N_5_ and N_10_ contain only low levels of incorporated DMAEMA, no significant alteration in polymer conformation is anticipated for these samples. The brush heights of the synthesized CBPs were determined from transmission electron microscopy images (TEM, Figure S1). Subsequently, the scaling factors for each composition were calculated (Figure 1 (a)). A decrease in scaling factor with increasing incorporation of DMAEMA was observed (Figure 1 (b)-(c)). N_20_ and N_25_ exhibited a scaling factor of 0.7. A further increase in DMAEMA incorporation for N_30_ and N_35_ resulted in a reduction of the scaling factor to 0.6 and 0.4, respectively. With values lower than 0.9 for N_0_, increased chain mobility and, consequently, enhanced entanglement formation was anticipated for these samples.

**Figure 1:**
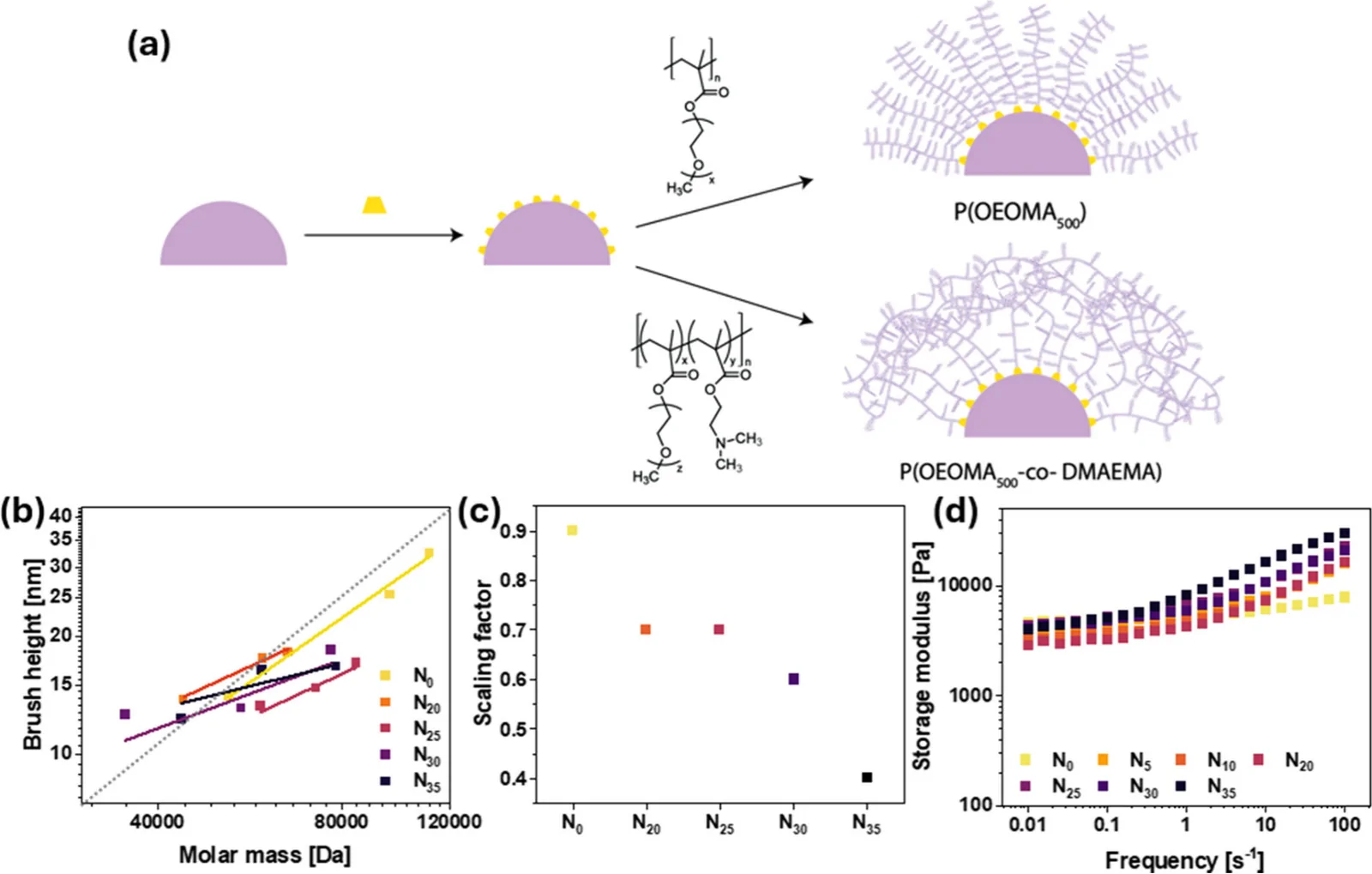
Schematic illustration of functionalization of SiO_2_ nanoparticles and polymer conformation after subsequent grafting of bulky (OEOMA_500_) vs less bulky (DMAEMA) monomers (a), dependence of brush height on degree of polymerization (b), relationship between scaling factor and DMAEMA incorporation (c) and storage moduli determined in frequency sweeps from 0.01 to 100 s^–1^ at room temperature (d).

Although DMAEMA contains tertiary amino groups capable of interacting with ionic species, significant protonation of these groups is not expected under the aprotic, anhydrous conditions of LiTFSI-based electrolytes. In the absence of protic media, the p*K*_a_-governed protonation equilibrium that drives polyelectrolyte brush expansion is effectively quenched, and the DMAEMA units are anticipated to behave predominantly as neutral polymer segments. It is nonetheless acknowledged that tertiary amines can engage in weak Lewis acid–base coordination with Li^+^. Therefore, they can potentially form transient amino-Li^+^ complexes that compete with the ether oxygen coordination sites characteristic of PEO-based systems. However, the donor number of aliphatic tertiary amines compared to ether oxygens in aprotic media is relatively low, while the LiTFSI concentration employed here is high, which substantially screens electrostatic interactions through ion pairing. Hence, any such coordination is expected to be minor and unlikely to drive the electrostatic brush expansion characteristic of charged polyelectrolyte systems. Consequently, the conformational behavior of the DMAEMA-containing brushes in this work is interpreted as a steric rather than an electrostatic effect. Here, changes in brush conformation attributed to the increased flexibility and reduced packing density of the tertiary amino-containing repeat units relative to their more sterically constrained counterparts.

### 2.2 Mechanical Testing

If coiled chain conformations enable entanglement, increasing DMAEMA content should produce measurably stiffer CPB films. Mechanical properties of the synthesized CBP samples were evaluated by frequency sweep and creep testing using dynamic mechanical analysis (Figures 1 (d) and S2). Storage and loss moduli determined in the frequency sweep showed increased values compared to N_0_ at high frequencies (Figures 1 (d) and S2 (a)). In addition, higher moduli were obtained for the highest DMAEMA content samples, N_30_ and N_35_, at high frequencies compared to the residual DMAEMA containing CBPs. These observations were ascribed to the combined effects of higher *T*_g_ of the incorporated DMAEMA and the increasing formation of entanglements resulting from the incorporation of a monomer with smaller functional groups. Creep tests showed the highest maximum shear strain for N_0_ with 10.5% (Figure S2 (b)). The CBP materials containing DMAEMA exhibited a decrease in the maximum shear strain with increasing DMAEMA content, reaching 1.8% for N_35_. Owing to the higher *T*_g_ of DMAEMA, increased incorporation ratios resulted in stiffer materials, resulting in lower elasticity at room temperature.

### 2.3 Electrochemical Testing

Ionic conductivity of the CBP materials mixed with LiTFSI (EO:Li = 10:1) was measured by electrochemical impedance spectroscopy at temperatures ranging from 25 to 60 °C (Figure 2 (a)). With ionic conductivities of 6.4 × 10^–6^ S cm^–1^ at room temperature and 4.1 × 10^–5^ S cm^–1^ at 60 °C, N_0_ showed the highest values of the tested CBPs. Increasing the DMAEMA incorporation ratio resulted in a decrease in ionic conductivity, with the lowest values observed for N_35_ with 1.9 × 10^–7^ S cm^–1^ and 4.7 × 10^–6^ S cm^–1^ at room temperature and 60 °C, respectively. While the amino groups of DMAEMA have been shown to stabilize the lithium interface, they provide limited ionic conductivity compared to OEOMA_500_. Hence, as the EO units are the major contributors to lithium transport through the material, the ionic conductivity decreased upon lowering the incorporation ration of OEOMA_500_. Additionally, because the EO:Li ratio was maintained at 10:1 while the OEOMA_500_ content decreased, the overall LiTFSI concentration correspondingly declined with increasing DMAEMA incorporation. It is well established that a reduction in lithium salt concentration leads to diminished ionic conductivity. Therefore, the observed decrease in ionic conductivity of the CBP materials was attributed to the combined effect of a reduced fraction of ionically conductive segments and a lower salt content. Although these ionic conductivity values are lower than typical polymeric electrolytes, they are sufficient for their intended application as an aSEI. In this case, the thin interfacial nature of the coating minimizes ion transport distances while prioritizing interfacial stability and mechanical protection of the lithium–metal surface. The reduction in ionic conductivity with simultaneous increase in mechanical properties reveals the characteristic trade-off between mechanical stiffness and ion transport. When entanglements are introduced, chain mobility is diminished leading to hampered ionic conductivity.

**Figure 2:**
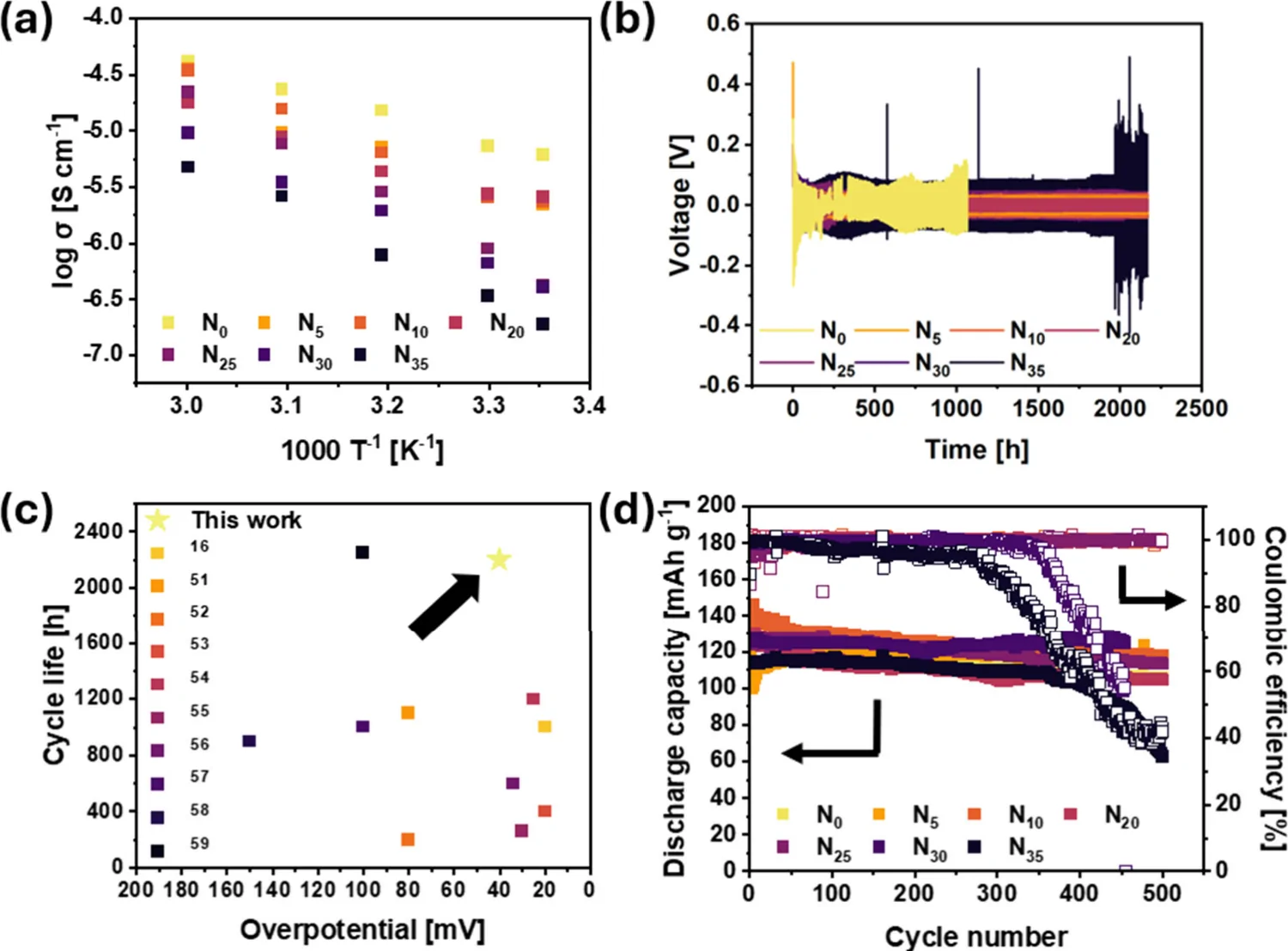
Ionic conductivity of CBP materials mixed with LiTFSI (EO:Li = 10:1) at various temperatures from 25 to 60 °C (a), symmetric cycling of CBP@Li|CBP@Li cells at 1 mA cm^–2^ and 0.5 mAh cm^–2^ (b), Ashby-like plot comparing the performance in symmetric cells of this work to various references 16,51–59 (c) and cycling stability (discharge capacity with filled squares and Coulombic efficiency with hollow squares) of CBP@Li|LFP cells at 0.5C (d).

The CBP samples were evaluated as aSEIs in CBP@Li|CBP@Li symmetric cells at a current density of 1 mA cm^–2^ and an areal capacity of 0.5 mAh cm^–2^ (Figure 2 (b)). Additionally, Li|Li was tested as a control (Figure S3 (a)). The cycling showed unstable performance with increased overpotentials up to 500 mV and shorting after 1550 h. The symmetric cell employing N_0_ as the aSEI failed after approximately 1100 h due to short-circuiting, accompanied by unstable cycling and an elevated overpotential of up to 150 mV during its cycle life. In contrast, N_5_ – N_25_ demonstrated stable cycling for over 2000 h with overpotentials in the range of 20 – 50 mV, with N_5_ showing the lowest values. N_30_ showed higher voltages in the first 400 cycles up to 100 mV. At higher cycle numbers, however, overpotentials stabilized at ∼ 60 mV providing stable cycling. Further increasing the DMAEMA incorporation ratio of the aSEI to 35 wt % resulted in an increase of the overpotential to 300 mV at the end of the cycle life, as well as unstable cycling behavior (Figure S4). The deterioration in cycling performance observed in the symmetric cells was ascribed to the combined effects of decreased ionic conductivity and compromised mechanical properties at higher DMAEMA contents.

To further contextualize the electrochemical performance, an Ashby-like plot comparing cycle life and corresponding overpotentials in symmetric cells was constructed (Figure 2 (c)). The literature data in the Ashby-like plot are drawn from sources with broadly similar, though not identical, testing conditions. As such, the comparisons shown should be interpreted as indicative rather than strictly equivalent. Literature data for composite aSEIs revealed a characteristic trade-off. While extended cycling stability is typically accompanied by elevated overpotentials, low overpotentials are often associated with limited cycle life. In contrast, the CBP-based aSEIs, particularly N_5_–N_25_, were found to exhibit prolonged cycle life exceeding 2000 h in combination with low overpotentials as low as 40 mV. This behavior highlighted the ability of the CBP architecture to mitigate the conventional trade-off between interfacial stability and ionic transport.

The surface morphologies of the lithium anodes after 2000 cycles were examined via scanning electron microscopy (SEM, Figure S5). Bare Li and N_0_ exhibited nonuniform lithium deposition with observable dendrite growth, which resulted in short-circuiting of the cell (Figure S5 (a) - (c)). Incorporation of low amounts of DMAEMA in N_5_ – N_25_ led to significantly smoother surface morphologies after cycling, indicating uniform lithium deposition (Figure S5 (d) – (g)). Further increases in DMAEMA content in N_30_ and N_35_ resulted in rougher surface morphologies and cracking showing a different failure mechanism than N_0_ (Figure S5 (h) + (i)). The nonuniform lithium deposition, as well as the surface cracking, was ascribed to the combined effects of decreased ionic conductivity and reduced elasticity.

Rate capability measurements were carried out in CBP@Li|lithium iron phosphate (LFP) and Li|LFP full cells at C-rates of 0.1, 0.2, 0.5, 1, and 2 (Figure S6). All tested cells exhibited comparable decreases in discharge capacity with increasing C-rates. In addition, high reversibility was observed when C-rate decreased to 0.1 in the last testing phase. To enable an efficient comparison of rate performance, the capacity loss from 0.1 to 2 C-rate was determined for each cell. For N_0_, a capacity loss of 37.0% was obtained. Incorporation of a small amount of DMAEMA in N_5_ resulted in a slight decrease in capacity loss to 29.2%, while a further increase in DMAEMA content led to capacity losses of 39.3% and 45.9% for N_10_ and N_20_. With additional increases in DMAEMA incorporation, the capacity loss initially decreased to 22.7% and subsequently increased from 28.7% to 31.1% for N_25_, N_30_, and N_35_, respectively. Due to the lower ionic conductivity at the highest DMAEMA ratios, lithium-ion transport was limited, which became evident at higher C-rates.

CBP materials were further tested by cycling for 500 cycles in CBP@Li|LFP at 0.5 C-rate following 5 initiation cycles at 0.1 C-rate (Figure 2 (d)). Bare lithium was tested as a control and showed stable cycling with a capacity retention of 83% after 500 cycles as well as Coulombic efficiency of >95%. (Figure S3 (b)). Application of N_0_ as the aSEI resulted in a continuous decrease in specific discharge capacity, yielding a capacity retention of 78%. In contrast, N_5_ – N_25_ employed as aSEIs provided stable performance over 500 cycles and mostly outperformed bare lithium, with capacity retentions ranging from 83 to 97%. For N_30_ and N_35_, however, a pronounced loss in both discharge capacity and Coulombic efficiency was observed. While the discharge capacity remained stable for N_30_ with a capacity retention of 95%, the Coulombic efficiency decreased substantially after approximately 375 cycles to 55% at the end of its cycle life. N_35_ exhibited a decrease in Coulombic efficiency after approximately 300 h. The discharge capacity did not show a significant decline until approximately 400 cycles, resulting in a capacity retention of 55%. Hence, applying these aSEIs resulted in a significant deterioration in electrochemical performance.

To further evaluate the origin of the observed performance decline, voltage profiles for selected cycle numbers ranging from 5 – 400 cycles were plotted for N_25_, N_30_ and N_35_ (Figure 3 (a)-(c)). N_25_ exhibited stable cycling with low polarization, consistent with the steady cycling behavior observed previously. In contrast, N_30_ and N_35_ showed potential fluctuations and a pronounced increase in charge capacity at the 400th cycle, increasing from 125 mAh g^–1^ to 175 mAh g^–1^ and from 115 mAh g^–1^ to 165 mAh g^–1^, respectively. In addition, the discharge capacity of N_35_ decreased at the 400th cycle from 115 mAh g^–1^ to 102 mAh g^–1^. These discrepancies between charge and discharge capacities indicated the occurrence of soft shorting in these cells.

**Figure 3:**
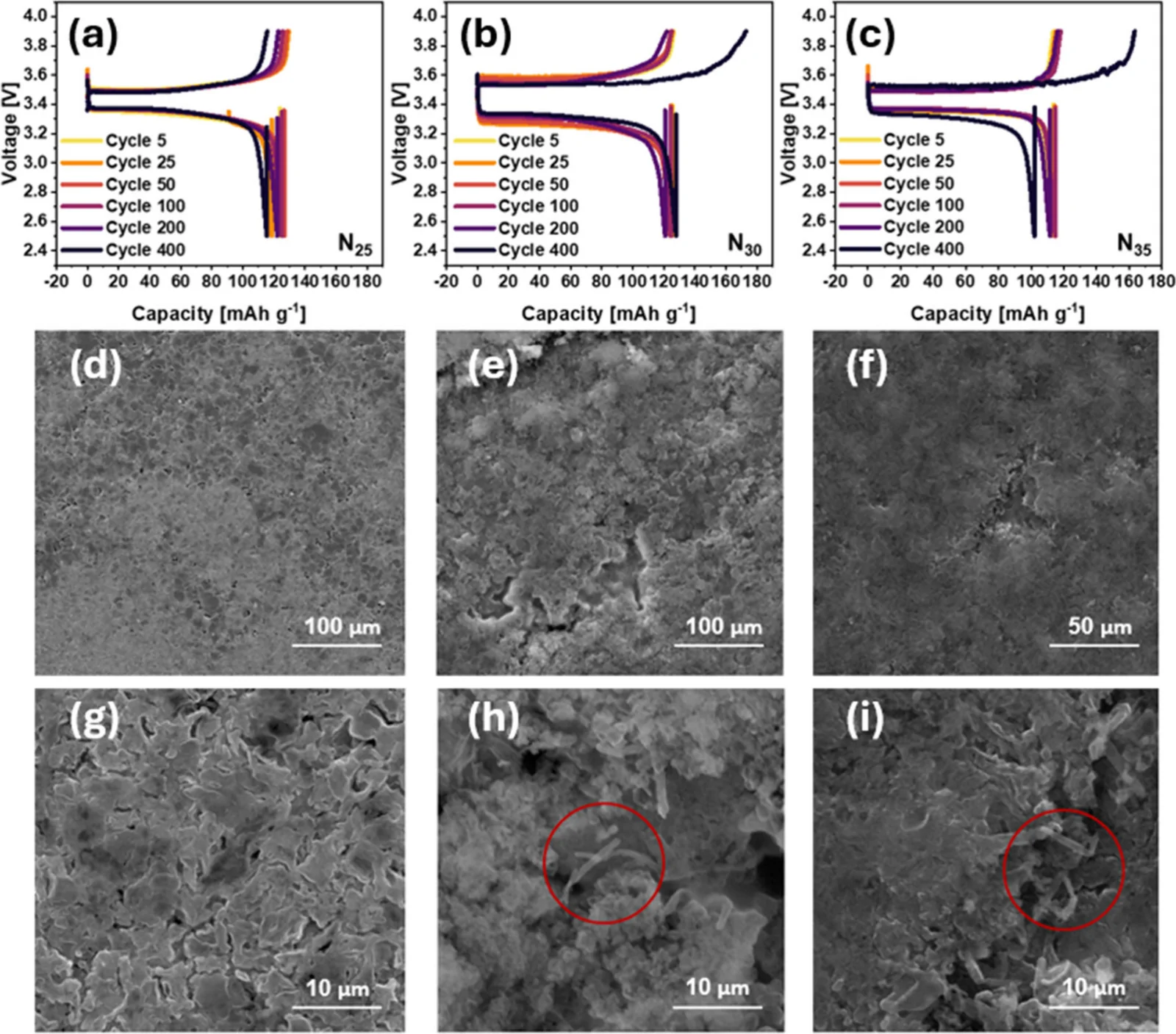
Voltage curves (a-c) for CBP@Li cells of the 5th, 25th, 50th, 100th, 200th and 400th cycle for N_25_ (a), N_30_ (b) and N_35_ (c) and SEM images (d-i) of Li anodes protected by N_25_ (d)+(g), N_30_ (e)+(h), and N_35_ (f)+(i) with dendrite formation (circled red).

The surface morphology of the lithium anode after 500 cycles was examined by scanning electron microscopy (SEM, Figure 3 (d)-(i)). A smooth surface morphology was found for N_25_ (Figure 3 (d) and (g)). In contrast, N_30_ and N_35_ exhibited mossy lithium growth, indicating nonuniform lithium deposition during cycling (Figure 3 (e) and (h), and (f) and (i)). Furthermore, lithium dendrites were observed, supporting the explanation of the inferior electrochemical performance of N_30_ and N_35_ due to soft shorting.

It is acknowledged that increasing DMAEMA incorporation into the brush architecture simultaneously modifies several material parameters beyond brush conformation alone. Hence, attributing the observed improvements in storage modulus and electrochemical performance for N_5_–N_25_ exclusively to enhanced chain entanglement would represent an oversimplification. The substitution of OEOMA repeat units with DMAEMA reduces local density of ethylene oxide-based Li^+^-solvating segments, which in isolation would be expected to diminish ionic conductivity by reducing the number of available coordination sites per chain. Concurrently, the incorporation of DMAEMA alters the glass transition temperature of the brush layer. As mentioned previously, DMAEMA exhibits a higher *T*_g_ value than OEOMA due to reduced side-chain plasticization, leading to diminished ionic conductivities. The fact that electrochemical performance nonetheless improved with increasing DMAEMA content therefore suggests that the conformational and topological changes at the brush level outweigh the previously mentioned opposing contributions.

### 2.4 Crosslinked aSEI

Physical entanglements are dynamically reversible as they disengage under stress, permitting creep and heterogeneous swelling of the aSEI film. Chemical crosslinks provide permanent reinforcement, but ion transport pathways have to be maintained by controlling crosslinking density. The incorporated DMAEMA provided opportunities for crosslinking via quaternization of amino groups. CBPs with high GD and low molar mass exhibited inconsistent swelling due to reduced entanglements.^33^ This can be mitigated by applying a thin crosslinked layer on top of the aSEI to ensure even swelling of the CBP material. 1,6-Dibromohexane and 1,4-butaneditriflate were chosen as crosslinkers to evaluate the effect of the leaving group/counterion on the performance (Figure 4 (a)). Alkyl triflates react significantly faster in a Menshutkin reaction, leading to a direct correlation between crosslinking kinetics and battery performance in this study. Here, only N_5_ – N_20_ were tested to ensure sparse crosslinking due to the ionic nature of the crosslinking. The aSEIs were prepared by drop casting the CBP materials on lithium chips and drying for 1h at 65 °C. After returning to room temperature, the crosslinker in an acetone solution was added. The temperature was held at room temperature for 30 min and, subsequently, the substrate was dried for 2h at 65 °C before cell assembly (Figure 4 (b)).

**Figure 4:**
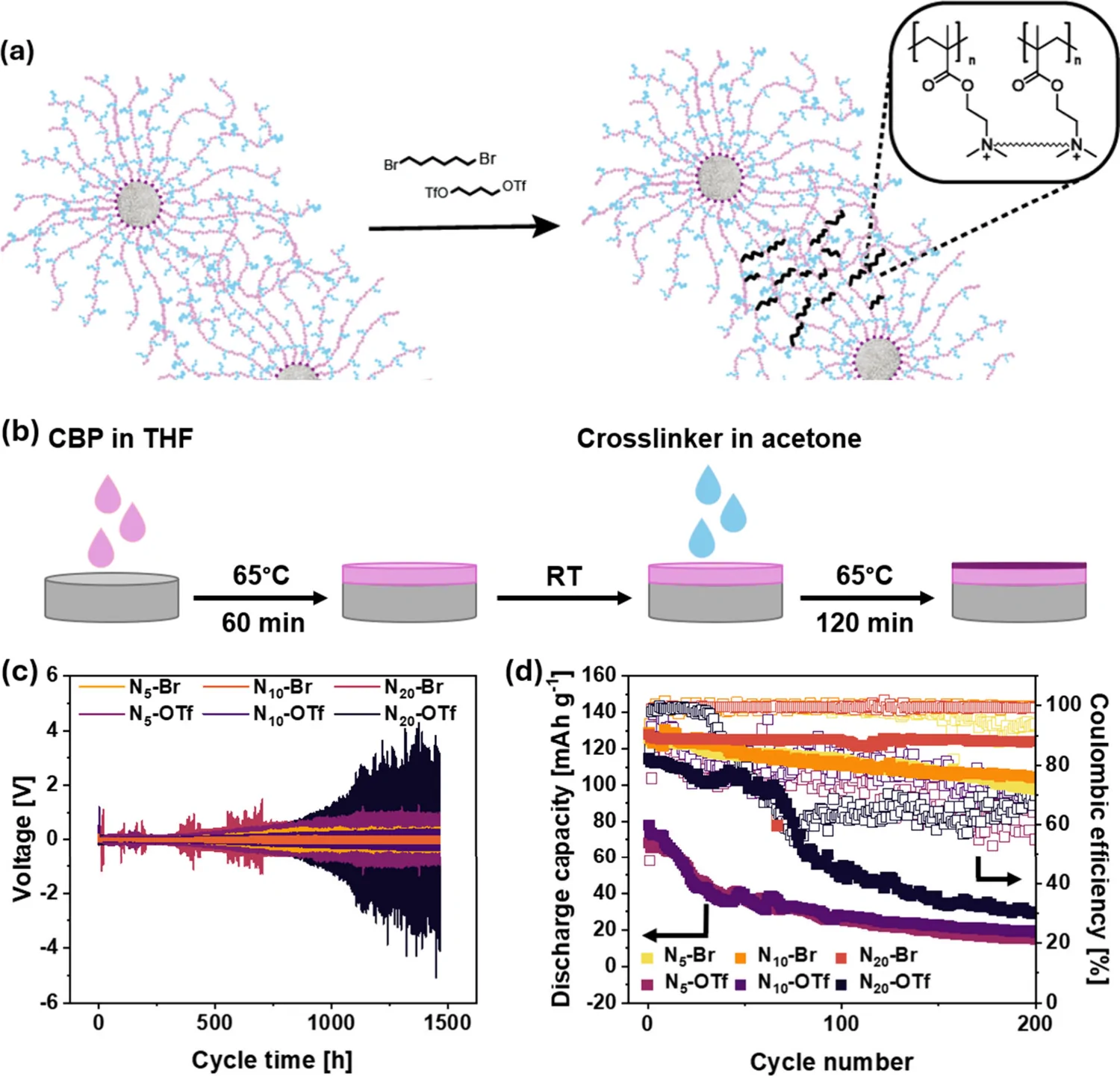
Schematic illustrations of CBP crosslinking (a) and Li electrode preparation before cell assembly (b), symmetric cycling of crCBP@Li|crCBP@Li cells at 1 mA cm^–2^ and 0.5 mAh cm^–2^ (c) and cycling stability (discharge capacity with filled squares and Coulombic efficiency with hollow squares) of crCBP@Li|LFP cells at 0.5C (d).

The crosslinked aSEIs were evaluated in symmetric crCBP@Li|crCBP@Li cells at a current density of 1 mA cm^–2^ and an areal capacity of 0.5 mAh cm^–2^ (Figure 4 (c)). A significant performance divergence was revealed between the two crosslinkers. The use of 1,6-dibromohexane as the crosslinker provided stable cycling with the lowest observed overpotentials of 110 mV for N_10_. For N_5_ and N_20_, increased overpotentials of up to 460 mV and 1.48 V were observed, with unstable cycling for N_20_. In contrast, 1,4-butaneditriflate, as the crosslinker, resulted in significantly higher overpotentials than 1,6-dibromohexane. While overpotentials of 380 mV were observed for N_10_, N_5_ and N_20_ exhibited increased overpotentials after 1100 h, reaching up to 1.17 and 4.29 V, respectively. Overall, higher overpotentials were observed compared to CBP samples with no crosslinking.

Surface morphologies of the lithium anodes after cycling were examined by SEM (Figure S7). All anodes protected by a CBP layer, topically crosslinked with 1,6-dibromohexane, exhibited smooth surfaces, indicating uniform lithium deposition (Figure S7 (a) – (c)). On the other hand, the anodes protected by CBPs crosslinked using 1,4-butaneditriflate exhibited an increased amount of ‘dead’ lithium and surface cracking (Figure S7 (d) – (f)).

Rate capability measurements were performed in crCBP@Li|LFP cells at various C-rates ranging from 0.1 to 2 (Figure S8). Using 1,6-dibromohexane as the crosslinker resulted in high discharge capacities exceeding 130 mAh g^–1^ at 0.1 C-rate and 80 mAh g^–1^ at 2 C-rate, as well as good reversibility upon returning to 0.1 C-rate. In contrast, CBPs crosslinked using 1,4-butaneditriflate exhibited reduced discharge capacities of approximately 110 mAh g^–1^ at 0.1 C-rate and ranging from 0.5 to 50 mAh g^–1^ at 2 C-rate.

Furthermore, crCBP@Li|LFP cells were cycled at a 0.5 C-rate for 200 cycles (Figure 4 (d)). Crosslinked CBPs using 1,6-dibromohexane demonstrated stable cycling and high Coulombic efficiencies, with capacity retentions of 78%, 81% and 97% for N_5_, N_10_ and N_20_, respectively. Crosslinking the CBPs using 1,4-butaneditriflate, however, resulted in a significant reduction in the initial specific discharge capacity to 65 mAh g^–1^ for N_5_ and 75 mAh g^–1^ for N_10_. Additionally, notably low capacity retentions of 22% and 24% were observed. N_20_ exhibited an initial specific discharge capacity of 114 mAh g^–1^, comparable to that obtained for 1,6-dibromohexane. However, a significant loss in specific discharge capacity and Coulombic efficiency was observed, resulting in a capacity retention of 26%. While the cycling window of 200 cycles is acknowledged as a limitation, the premature capacity fade observed in combination with the increased overpotentials in symmetric cycling called for early test termination.

The reduced performance of the protective layers crosslinked using 1,4-butaneditriflate compared to 1,6-dibromohexane was ascribed to the differences in the kinetics of the quaternization reaction and the resulting differences in crosslink density and ionic conductivity. Owing to the inferior leaving group, bromides exhibited slower rates in the Menshutkin reaction in comparison to triflates (Figure S9). Topical application of the crosslinker resulted in a smaller number of crosslinks per layer for bromides due to the slower reaction (Figure 5 (a)). Here, a lower crosslink density was produced which maintained more accessible pathways for lithium-ion transport. In contrast, triflates react within seconds, leading to a substantial increase in the number of crosslinks per layer within the CBP material (Figure 5 (b)). Owing to the ionic nature of crosslinks, an increased crosslink density restricted the available pathways of the lithium-ion transport throughout the material, leading to the reduced ionic conductivity. Consequently, the battery performance was adversely affected when 1,4-butaneditriflate was employed as the crosslinker due to the diminished ionic conductivity arising from the higher crosslink density. Hence, bromide crosslinkers were chosen for further investigation.

**Figure 5:**
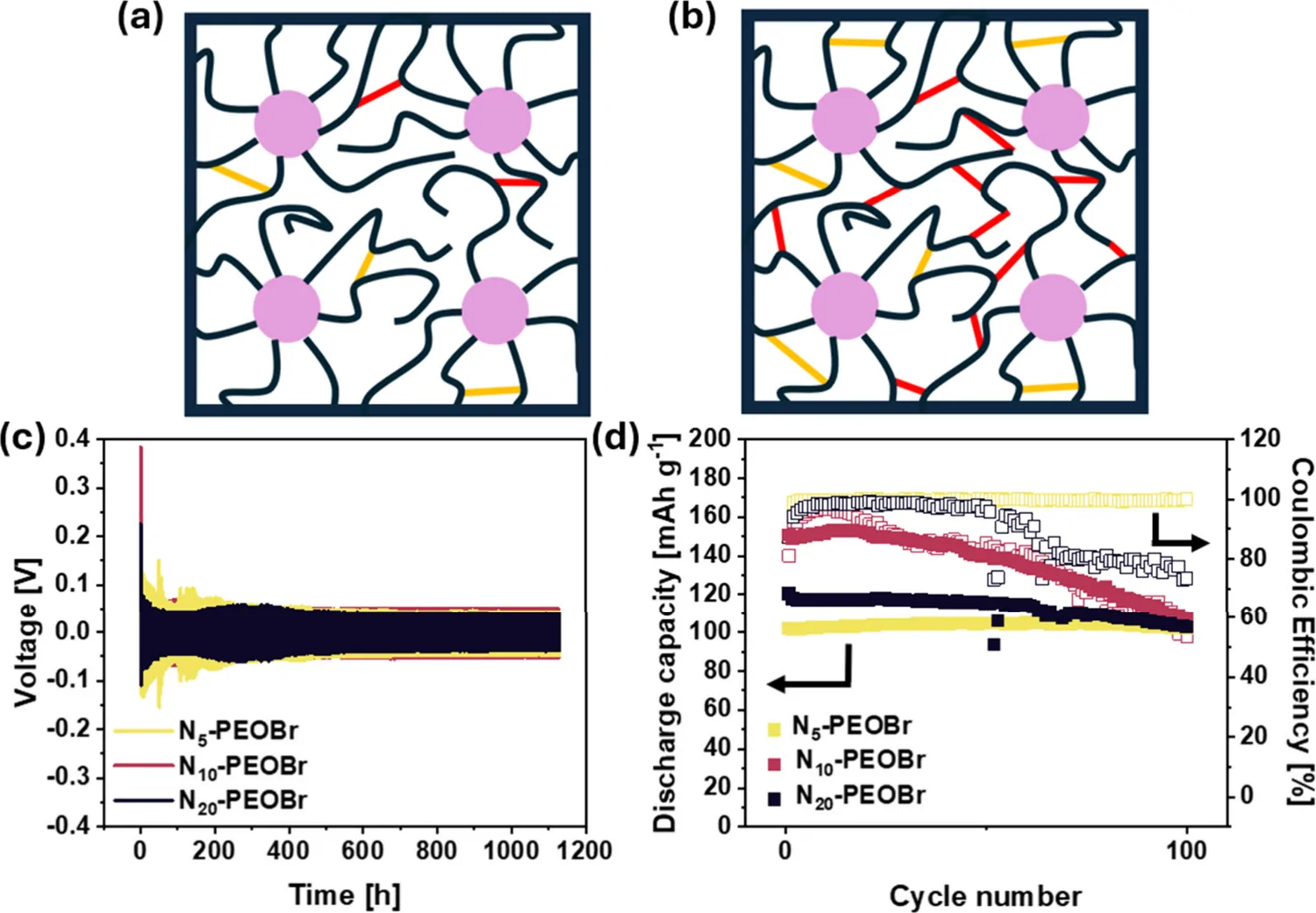
Schematic illustration of crosslinked CBPs showing interparticle (red) and intraparticle (yellow) crosslinks using a bromide crosslinker (a) and using a triflate crosslinker (b), symmetric cycling of crCBP@Li|crCBP@Li cells using PEOBr as the crosslinker at 1 mA cm^–2^ and 0.5 mAh cm^–2^ (c) and cycling stability (discharge capacity with filled squares and Coulombic efficiency with hollow squares) of crCBP@Li|LFP cells using PEOBr as the crosslinker at 0.5C (d).

Due to the short segment lengths between crosslinking sites for 1,6-dibromohexane and 1,4-butaneditriflate, these compounds are prone to facilitating intraparticle crosslinks (Figure 5 (a) and (b)). These intraparticle crosslinks do not contribute to maintaining an even swelling throughout the CBP material. To enable interparticle crosslinks and take advantage of the benefits of the bromide counterion, Br-PEO_2k_-Br was synthesized and evaluated as a longer-chain crosslinker.^60^

Symmetric cycling was performed in crCBP@Li|crCBP@Li cells at a current density of 1 mA cm^–2^ and areal capacity of 0.5 mAh cm^–2^ (Figure 5 (c)). While N_5_ exhibited slightly increased overpotentials up to 150 mV during the initial 200 cycles, the cell stabilized at 45 mV for the residual testing time. N_10_ and N_20_ showed stable cycling with negligible variances and overpotentials of 50 mV and 40 mV, respectively. Surface morphologies were examined by SEM (Figure S10). Smooth surfaces were observed for all tested CBPs, indicating uniform lithium deposition during cycling.

Rate capability were measured in crCBP@Li|LFP cells at C-rates ranging from 0.1 to 2 (Figure S11). While N_5_ showed reduced discharge capacities of 115 mAh g^–1^ at 0.1 C-rate and 70 mAh g^–1^ at 2 C-rate, good reversibility was observed when a C-rate was applied at the end of testing. Using N_10_ as the crosslinked protective layer resulted in a high initial discharge capacity of 135 mAh g^–1^ in the first cycle at 0.1 C-rate. However, a significant decrease in discharge capacity of 15 mAh g^–1^ was observed within the 5 cycles at 0.1 C-rate. Further decrease to 40 mAh g^–1^ at 2 C-rate as well as poor reversibility to 90 mAh g^–1^ at 0.1 C-rate indicated passivation of the lithium surface. In contrast, N_20_ exhibited high discharge capacity of 150 mAh g^–1^ at 0.1 C-rate and 100 mAh g^–1^ at 2 C-rate as well as good reversibility at 0.1 C-rate.

Additionally, crCBP@Li|LFP cells were cycled for 100 cycles at 0.5 C-rate (Figure 5 (d)). While N_5_ showed stable cycling at reduced discharge capacity of 100 mAh g^–1^ with high Coulombic efficiency and capacity retention of 99%, N_10_ and N_20_ exhibited inferior cycling stability. Decreasing Coulombic efficiencies were observed after 10 and 50 cycles for N_10_ and N_20_, respectively. The accompanying decrease in discharge capacities led to capacity retentions of 71% and 85% and indicated soft shortening of the battery cells.

To examine the surface morphologies after 100 cycles, SEM was performed (Figure S12). A smooth anode surface was observed after the protection with the crosslinked N_5_ using Br-PEO_2k_-Br. N_10_ and N_20_, however, presented rougher surfaces, indicating nonuniform lithium deposition, which hampered cycling performance.

## 3 Experimental Section

### 3.1 Materials

Silica nanoparticles (average core diameter d = 15.8 nm, supplied as a 30 wt % solution in methyl ethyl ketone) were received from Nissan Chemicals and used without further purification. The surface initiator, 3-(chlorodimethylsilyl)­propyl α-bromoisobutyrate, was prepared according to a previously reported procedure.^47^ The syntheses of 1,4-butaneditriflate and Br-PEO-Br were adapted from a previously reported route.^60,61^ Oligo­(ethylene oxide) methyl ether methacrylate with an average molecular mass of 500 g mol^–1^ (OEOMA_500_, Sigma-Aldrich) and 2-(dimethylamino)­ethyl methacrylate (DMAEMA, Sigma-Aldrich) were passed through a column of basic alumina prior to use in order to remove inhibitor. The following reagents were used as received unless otherwise specified: 1,6-Dibromohexane (Sigma-Aldrich, 96%), 1,4-butanediol (Sigma-Aldrich), triflic anhydride (Sigma-Aldrich, 99%), poly­(ethylene oxide) (average *M*_n_ = 4600 g mol^–1^, hydroxyl, Sigma-Aldrich), 4-toluenesulfonyl chloride (Sigma-Aldrich), triethylamine (Sigma-Aldrich, ≥ 99%), sodium bromide (Sigma-Aldrich, ≥ 99%), copper bromide (CuBr_2_, Sigma-Aldrich, 99.9%), tris­(2-dimethylaminoethyl)­amine (Me_6_TREN, Ambeed, 99%), tin­(II) 2-ethylhexanoate (Sn­(EH)_2_, Sigma-Aldrich, 95%), 48 wt % aqueous hydrofluoric acid (HF, 99.99%, Sigma-Aldrich), alumina (basic, Super I, 50–200 μm, Sorbtech), lithium bis­(trifluoromethane)­sulfonimide (LiTFSI, Sigma-Aldrich, anhydrous, 99.99%), lithium nitrate (LiNO_3_, 99.99% Sigma-Aldrich), anisole (Thermo Fisher, 99%), N,N-dimethylformamide (DMF, Thermo Fisher, 99.8%), hexane (Thermo Fisher), methanol (MeOH, Thermo Fisher), tetrahydrofuran (THF, Sigma-Aldrich, 99.5%), acetone (Thermo Fisher), dichloromethane (Thermo Fisher), diethyl ether (Thermo Fisher, anhydrous), dimethyl sulfoxide (DMSO, Thermo Fisher), 1,3-dioxolane (DOL, Sigma-Aldrich, anhydrous, 99.5%), 1,2-dimethoxyethane (DME, Sigma-Aldrich, anhydrous, 99.5%) and 1-methyl-2-pyrrolidinone (NMP, Sigma-Aldrich, anhydrous, 99.5%).

### 3.2 CBP Synthesis

Surface functionalization of the silica nanoparticles with 3-(chlorodimethylsilyl)­propyl α-bromoisobutyrate was conducted as previously described.^47^

For polymer brush growth, the functionalized silica nanoparticles were dispersed in a solution containing OEOMA_500_, DMAEMA, anisole (1:1 v/v) with DMF (1 mL) added as cosolvent. CuBr_2_ (200 ppm) and Me_6_TREN ([Cu]/[ligand] = 1/3) were introduced. The reaction mixture was transferred to a dried Schlenk flask and sealed. Dissolved oxygen was removed by nitrogen sparging for 20 min. Following deoxygenation, the flask was immersed in an oil bath maintained at 50 °C. Sn­(EH)_2_ ([Cu]/[Tin] = 1/5) was added dropwise. The reaction was allowed to proceed at 50 °C under stirring until a viscous dispersion was obtained. The resulting CBPs were isolated by precipitation into hexane. Further purification was achieved by dialysis in MeOH (1 cycle) and THF (2 cycles).

### 3.3 Analysis

To determine the molar mass of the grafted polymers, silica cores were removed by etching with HF. For that, CBPs (20 mg) were dispersed in THF (10 mL) and HF (1 mL) was added dropwise. The solution was left stirring for 12 h. The reaction mixture was neutralized with ammonia. The polymer solution was passed through neutral alumina followed by filtration through a 0.45 μm PTFE filter. Gel permeation chromatography (GPC) measurements were performed in THF at 35 °C using an Agilent system equipped with Polymer Standards Services (PSS) columns (guard, 10^5^, 10^3^ and 10^2^ Å) and a Waters 2410 differential refractive index detector. The flow rate was maintained at 1.00 mL min^–1^. Number-average molecular mass (*M*_n_) and molecular weight dispersity (*M*_w_/*M*_n_) were calculated from the resulting chromatograms.

Scanning electron microscopy (SEM) analysis of the lithium electrode surfaces was conducted using a Tescan Mira 3. Lithium anodes were extracted from the coin cells post cycling and measured without further treatment.

Transmission electron micrscopy (TEM) was performed on a FEI Tecnai F20 Super-Twin electron microscope operated at 200 kV to evaluate interparticle spacing. Samples were prepared by drop casting CBP dispersions from a THF solution onto carbon-coated copper grid prior to imaging.

Thermogravimetric analysis (TGA) was carried out using a TA Instruments 2950 to quantify the inorganic silica fraction within the CBPs. The thermal program consisted of heating at 30 °C min^–1^ to 120 °C, holding at 120 °C for 10 min followed by heating at 20 °C min^–1^ to 800 °C. Data analysis was conducted using TA Universal Analysis software.

The scaling factor was estimated by correlating the brush height from TEM images as a function of *M*_n,abs_. The TEM images are shown in Figure S1. Brush height was calculated by subtracting the measured core-to-core particle distance with each core radius and divided by two.

Rheological measurements were performed using an Anton Paar MCR-302 Rheometer equipped with a parallel plate geometry. CBP films were prepared by drop casting from a THF solution directly on the lower plate, and a nominal force of 1N was applied. Frequency sweep experiments were conducted at room temperature over the range 0.01 to 100 s^–1^ to obtain the storage and loss moduli. Creep experiments were carried out by applying a shear stress of 100 Pa for 100 s, followed by 0 Pa for 75 s.

CR2032-type coin cells were assembled inside an argon-filled glovebox with H_2_O and O_2_ levels maintained below 0.5 ppm. All electrochemical tests were performed at room temperature unless otherwise specified.

AC impedance spectroscopy was used to determine ionic conductivity. CBP materials blended with LiTFSI at an EO:Li ratio of 10:1 were drop cast into a Teflon washer and sandwiched between stainless steel plates (Stainless steel|CBP/LiTFSI|Stainless steel). Measurements were conducted from 25 to 60 °C, with a 15 min equilibration period at each temperature prior to data acquisition. Ionic conductivity (σ) was calculated using eq 2.

2 $$\sigma =\frac{L}{R\cdot A}$$

L: the thickness of the CBP material, A: area of the material and R: charge-transfer resistance determined from the Nyquist plots.

Lithium iron phosphate (LFP) cathodes were fabricated from a slurry consisting of 85 wt % commercial LFP powder, 10 wt % Super-p, and 5 wt % polyvinylidene difluoride binder in NMP. The slurry was cast onto aluminum foil to achieve a mass loading of 2.5 mg cm^–2^. The liquid electrolyte consisted of 1 M LiTFSI dissolved in DOL/DME (1:1 v/v) containing 2 wt % LiNO_3_ to benefit from the combination of fast ion transport, low viscosity, high conductivity and SEI reinforcement. A volume of 60 μL electrolyte was used for each cell during assembly.

For aSEI preparation, 5 mg of CBP was drop cast from THF onto lithium metal chips (13 mm diameter and 0.6 mm thickness) and dried for 3 h at 65 °C.

For the crosslinked aSEI systems, a crosslinker solution (1,6-dibromohexane, 1,4-butaneditriflate or Br-PEO-Br in acetone, 50 μL of 1 mM solution in acetone) was applied at room temperature after one hour of drying. After evaporating the solvent at room temperature for 30 min, the samples were dried at 65 °C for two hours.

Symmetric cells were assembled using two CBP@Li electrodes and cycled at 1 mA cm^–2^ with a capacity of 0.5 mAh cm^–2^ to evaluate long-term lithium plating/stripping behavior. For Li|LFP full cells, CBP@Li anodes were paired with LFP cathodes. Rate capability was assessed at C-rates of 0.1, 0.2, 0.5, 1, and 2 for five cycles each. Long-term cycling consisted of six formation cycles at 0.1 C-rate followed by 100, 200, or 500 cycles at 0.5 C-rate.

## 4 Conclusion

In this study, comb-brush particles were rationally designed by incorporating to improve their application in artificial solid electrolyte interfaces. Incorporation of DMAEMA enabled systematic tuning of chain conformation and mechanical stiffness, revealing a direct correlation between reduced polymer chain extension, enhanced entanglement formation, and improved interfacial stability. However, excessive DMAEMA incorporation diminished ionic conductivity and promoted soft shorting. This emphasized the necessity of balancing mechanical reinforcement and ion transport. Optimal CBP compositions containing 5–25 wt % DMAEMA exhibited stable long-term cycling, uniform lithium deposition, and high capacity retention in full cells. Topical crosslinking further influenced battery performance, with bromide-based crosslinkers outperforming triflate analogs due to a lower crosslink density and preserved lithium-ion transport. Additionally, long-chain crosslinkers were examined to promote interparticle crosslinks and low overpotentials in symmetric cells. However, the diminished performance observed for higher DMAEMA incorporations in full cells indicated that further investigation and optimization of this approach are warranted. Overall, this work established entanglement and controlled ionic crosslinking as parameters for composite aSEIs, providing a versatile platform for stabilizing lithium–metal batteries through macromolecular engineering. These findings further underscore how deliberate macromolecular design can be used to rationally tailor electrochemical interfaces by balancing competing effects. Although increasing crosslink density enhances mechanical stiffness and suppresses plastic deformation and creep, it may simultaneously reduce toughness and promote embrittlement and cracking, while independently restricting ionic mobility. Accordingly, precise control over network architecture is essential to achieve synergistic optimization of mechanical robustness and ion transport.

## Supplementary Material



## Data Availability

All data analyzed during the study are included in this article and will be made available upon reasonable request.

## Notes

Molecular information for additional samples for scaling factor analysis, TEM images, frequency sweeps, creep tests, cycling of bare Li, quaternization kinetics, SEM images and rate capability tests (PDF)

## References

[1] Frith J. T., Lacey M. J., Ulissi U. (2023). A non-academic perspective on the
future of lithium-based batteries. Nat. Commun..

[2] Xu W., Wang J., Ding F., Chen X., Nasybulin E., Zhang Y., Zhang J.-G. (2014). Lithium
metal anodes for rechargeable
batteries. Energy Environ. Sci..

[3] Shen X., Liu H., Cheng X.-B., Yan C., Huang J.-Q. (2018). Beyond lithium ion
batteries: Higher energy density battery systems based on lithium
metal anodes. Energy Storage Materials.

[4] Li S., Lorandi F., Wang H., Liu T., Whitacre J. F., Matyjaszewski K. (2021). Functional polymers for lithium metal
batteries. Prog. Polym. Sci..

[5] Lorandi F., Liu T., Fantin M., Manser J., Al-Obeidi A., Zimmerman M., Matyjaszewski K., Whitacre J. F. (2021). Comparative performance
of ex situ artificial solid electrolyte interphases for Li metal batteries
with liquid electrolytes. iScience.

[6] Li S., Lorandi F., Whitacre J. F., Matyjaszewski K. (2020). Polymer Chemistry
for Improving Lithium Metal Anodes. Macromol.
Chem. Phys..

[7] Deng W., Qiu B., Chen J., Li Z., Tang Y., Liu Z., Meng Y. S. (2026). Understanding the degradation complexity of ultrahigh-energy
lithium metal batteries. Nature Reviews Chemistry.

[8] Yang L., Hagh N. M., Roy J., Macciomei E., Klein J. R., Janakiraman U., Fortier M. E. (2024). Review—Challenges
and Opportunities in Lithium Metal Battery Technology. J. Electrochem. Soc..

[9] Wang M., Bai Y., Li J. (2026). Interfacial regulation
strategies and mechanisms of
electrolyte additives in lithium metal batteries. Journal of Energy Storage.

[10] Liu W., Liu P., Mitlin D. (2020). Review of Emerging Concepts in SEI Analysis and Artificial
SEI Membranes for Lithium, Sodium, and Potassium Metal Battery Anodes. Adv. Energy Mater..

[11] Ramasubramanian A., Yurkiv V., Foroozan T., Ragone M., Shahbazian-Yassar R., Mashayek F. (2020). Stability of Solid-Electrolyte Interphase (SEI) on
the Lithium Metal Surface in Lithium Metal Batteries (LMBs). ACS Applied Energy Materials.

[12] Vishweswariah K., Ningappa N. G., Bouguern M. D., Kumar M R A., Armand M. B., Zaghib K. (2025). Evaluation and Characterization
of
SEI Composition in Lithium Metal and Anode-Free Lithium Batteries. Adv. Energy Mater..

[13] Yang S.-J., Jiang F.-N., Hu J.-K., Yuan H., Cheng X.-B., Kaskel S., Zhang Q., Huang J.-Q. (2023). Life cycle safety
issues of lithium metal batteries: A perspective. Electron.

[14] Ma L., Kim M. S., Archer L. A. (2017). Stable Artificial Solid Electrolyte
Interphases for Lithium Batteries. Chem. Mater..

[15] Cheng Z., Chen Y., Shi L., Wu M., Wen Z. (2023). Long-Lifespan
Lithium Metal Batteries Enabled by a Hybrid Artificial Solid Electrolyte
Interface Layer. ACS Appl. Mater. Interfaces.

[16] Naren T., Kuang G.-C., Jiang R., Qing P., Yang H., Lin J., Chen Y., Wei W., Ji X., Chen L. (2023). Reactive Polymer
as Artificial Solid Electrolyte Interface for Stable Lithium Metal
Batteries. Angew. Chem., Int. Ed..

[17] Han B., Feng D., Li S., Zhang Z., Zou Y., Gu M., Meng H., Wang C., Xu K., Zhao Y., Zeng H., Wang C., Deng Y. (2020). Self-Regulated Phenomenon
of Inorganic Artificial Solid Electrolyte Interphase for Lithium Metal
Batteries. Nano Lett..

[18] Li S., Liu T., Yan J., Flum J., Wang H., Lorandi F., Wang Z., Fu L., Hu L., Zhao Y., Yuan R., Sun M., Whitacre J. F., Matyjaszewski K. (2020). Grafting polymer
from oxygen-vacancy-rich nanoparticles to enable protective layers
for stable lithium metal anode. Nano Energy.

[19] Kang D., Xiao M., Lemmon J. P. (2021). Artificial
Solid-Electrolyte Interphase
for Lithium Metal Batteries. Batteries &
Supercaps.

[20] Gao S., Sun F., Liu N., Yang H., Cao P.-F. (2020). Ionic conductive
polymers as artificial solid electrolyte interphase films in Li metal
batteries - A review. Mater. Today.

[21] Wu N.-L., Weng Y.-T., Li F.-S., Yang N.-H., Kuo C.-L., Li D.-S. (2015). Polymeric artificial
solid/electrolyte interphases for Li-ion batteries. Progress in Natural Science: Materials International.

[22] Naren T., Jiang R., Kuang G.-c., Zhou L., Chen L. (2024). Functional
Polymers as Artificial Solid Electrolyte Interfaces for Stabilizing
Lithium Metal Anode. ChemSusChem.

[23] Fan J., Ding S., Zeng X., Gao S., Wen Z., Zeng X., Sun R., Ren L. (2022). Gecko-inspired
adhesive
structures enable efficiently thermal conductance and vibration dissipation
in a highly mismatched system. Chemical Engineering
Journal.

[24] Golkaram M., Portale G., Mulder P., Maniar D., Faraji S., Loos K. (2020). Order-disorder transition
in supramolecular polymer combs/brushes
with polymeric side chains. Polym. Chem..

[25] Li C., Gunari N., Fischer K., Janshoff A., Schmidt M. (2004). New Perspectives
for the Design of Molecular Actuators: Thermally Induced Collapse
of Single Macromolecules from Cylindrical Brushes to Spheres. Angew. Chem., Int. Ed..

[26] Yan J., Bockstaller M. R., Matyjaszewski K. (2020). Brush-modified materials: Control
of molecular architecture, assembly behavior, properties and applications. Prog. Polym. Sci..

[27] Hui C. M., Pietrasik J., Schmitt M., Mahoney C., Choi J., Bockstaller M. R., Matyjaszewski K. (2014). Surface-Initiated Polymerization
as an Enabling Tool for Multifunctional (Nano-)­Engineered Hybrid Materials. Chem. Mater..

[28] Johari S. N. A. M., Tajuddin N. A., Hanibah H., Deraman S. K. (2021). A Review: Ionic
Conductivity of Solid Polymer Electrolyte Based Polyethylene Oxide. Int. J. Electrochem. Sci..

[29] Synytska A., Svetushkina E., Martina D., Bellmann C., Simon F., Ionov L., Stamm M., Creton C. (2012). Intelligent Materials
with Adaptive Adhesion Properties Based on Comb-like Polymer Brushes. Langmuir.

[30] Shimamoto H., Cheng C.-H., Kamitani K., Kojio K., Higaki Y., Takahara A. (2019). Nanocomposite Elastomers
Composed of Silica Nanoparticles
Grafted with a Comb-Shaped Copolymer Brush. Macromolecules.

[31] Hueckel T., Luo X., Aly O. F., Macfarlane R. J. (2023). Nanoparticle Brushes: Macromolecular
Ligands for Materials Synthesis. Acc. Chem.
Res..

[32] Dienemann L. L., Yin R., Liu T., Stejer A., Kempkes V., Li S., Zenyuk I. V., Matyjaszewski K., Panzer M. J. (2023). Hybrid Particle
Brush Coatings with Tailored Design for Enhanced Dendrite Prevention
and Cycle Life in Lithium Metal Batteries. ACS
Applied Energy Materials.

[33] Kempkes V., Liu T., Tarnsangpradit J., Li S., Bockstaller M. R., Whitacre J. F., Matyjaszewski K. (2025). Effect of
Bottlebrush Particle Architecture
on Their Efficiency as Protective Layers in Li-Metal Batteries. ACS Applied Polymer Materials.

[34] Wool R. P. (1993). Polymer
entanglements. Macromolecules.

[35] Kong D.-C., Yang M.-H., Zhang X.-S., Du Z.-C., Fu Q., Gao X.-Q., Gong J.-W. (2021). Control of Polymer Properties by
Entanglement: A Review. Macromol. Mater. Eng..

[36] Robertson N. J., Kostalik H. A. I. V., Clark T. J., Mutolo P. F., Abruña H. D., Coates G. W. (2010). Tunable High Performance Cross-Linked Alkaline Anion
Exchange Membranes for Fuel Cell Applications. J. Am. Chem. Soc..

[37] Kumari M., Douglin J. C., Dekel D. R. (2021). Crosslinked
quaternary phosphonium-functionalized
poly­(ether ether ketone) polymer-based anion-exchange membranes. J. Membr. Sci..

[38] Hwang U., Moon H., Park J., Jung H. W. (2024). Crosslinking and
Swelling Properties of pH-Responsive Poly­(Ethylene Glycol)/Poly­(Acrylic
Acid) Interpenetrating Polymer Network Hydrogels. Polymers.

[39] Călina I., Demeter M., Crăciun G., Scărişoreanu A., Mănăilă E. (2024). The Influence of the Structural Architecture
on the Swelling Kinetics and the Network Behavior of Sodium-Alginate-Based
Hydrogels Cross-Linked with Ionizing Radiation. Gels.

[40] Eckert K. M., Bonsen J., Hajnal A., Gmeiner J., Hasse J., Adrian M., Karsten J., Kißling P. A., Penn A., Fiedler B., Luinstra G. A., Smirnova I. (2025). Enhancing
swelling kinetics of pNIPAM lyogels: The role of crosslinking, copolymerization,
and solvent. Fluid Phase Equilib..

[41] Liu Y., Lin D., Yuen P. Y., Liu K., Xie J., Dauskardt R. H., Cui Y. (2017). An Artificial Solid
Electrolyte Interphase with High Li-Ion Conductivity,
Mechanical Strength, and Flexibility for Stable Lithium Metal Anodes. Adv. Mater..

[42] Yu Z., Cui Y., Bao Z. (2020). Design Principles of Artificial Solid Electrolyte Interphases
for Lithium-Metal Anodes. Cell Reports Physical
Science.

[43] Jiang G., Li K., Yu F., Li X., Mao J., Jiang W., Sun F., Dai B., Li Y. (2021). Robust Artificial Solid-Electrolyte
Interfaces with Biomimetic Ionic Channels for Dendrite-Free Li Metal
Anodes. Adv. Energy Mater..

[44] Wang C., Ren L., Ying C., Liu J., Zhong W.-H. (2024). An Amino Acid-Enabled
Separator for Effective Stabilization of Li Anodes. ACS Appl. Mater. Interfaces.

[45] Wang Q., Yao Z., Zhao C., Verhallen T., Tabor D. P., Liu M., Ooms F., Kang F., Aspuru-Guzik A., Hu Y.-S., Wagemaker M., Li B. (2020). Interface chemistry
of an amide electrolyte for highly reversible lithium metal batteries. Nat. Commun..

[46] Wang Z., Al Alwan B., Simon Ng K. Y. (2023). Multi-functions
of amino thiophenol
additives for shielding lithium metal anode in advanced Li-S battery. Materials Science and Engineering: B.

[47] Yan J., Pan X., Wang Z., Zhang J., Matyjaszewski K. (2016). Influence
of Spacers in Tetherable Initiators on Surface-Initiated Atom Transfer
Radical Polymerization (SI-ATRP). Macromolecules.

[48] Matyjaszewski K., Jakubowski W., Min K., Tang W., Huang J., Braunecker W. A., Tsarevsky N. V. (2006). Diminishing catalyst concentration
in atom transfer radical polymerization with reducing agents. Proc. Natl. Acad. Sci. U. S. A..

[49] Jakubowski W., Min K., Matyjaszewski K. (2006). Activators
Regenerated by Electron
Transfer for Atom Transfer Radical Polymerization of Styrene. Macromolecules.

[50] Choi J., Hui C. M., Schmitt M., Pietrasik J., Margel S., Matyjazsewski K., Bockstaller M. R. (2013). Effect
of Polymer-Graft Modification on the Order Formation in Particle Assembly
Structures. Langmuir.

[51] Liu H., Tao R., Guo C., Zhang W., Liu X., Guo P., Zhang T., Liang J. (2022). Lithiated halloysite nanotube/cross-linked
network polymer composite artificial solid electrolyte interface layer
for high-performance lithium metal batteries. Chemical Engineering Journal.

[52] Hu Z., Zhang S., Dong S., Li W., Li H., Cui G., Chen L. (2017). Poly­(ethyl α-cyanoacrylate)-Based Artificial
Solid Electrolyte Interphase Layer for Enhanced Interface Stability
of Li Metal Anodes. Chem. Mater..

[53] Kim M. S., Ryu J.-H., Deepika, Lim Y. R., Nah I. W., Lee K.-R., Archer L. A., Cho W. (2018). Langmuir-Blodgett artificial
solid-electrolyte interphases for practical lithium metal batteries. Nature Energy.

[54] Huo S., Zhang Y., He Y., Fan W., Hu Z., Bao W., Jing X., Cheng H. (2023). A Brush-like Li-Ion Exchange Polymer
as Potential Artificial Solid Electrolyte Interphase for Dendrite-Free
Lithium Metal Batteries. J. Phys. Chem. Lett..

[55] Kim S., Jeong Y. H., Won G., Jo M. S., Seo S., Kwon D.-S., Jeong D., Shim J. (2025). Architecture-Tunable
Bottlebrush Polymers as Artificial Solid Electrolyte Interphases for
Lithium Metal Batteries. Nano Lett..

[56] Meng Y., Yin R., Wu Y., Biao J., Kang G., Kang F., Cao Y. (2025). N,N-Dimethylacrylamide-mediated composite artificial solid electrolyte
interphase for long-cycling lithium metal batteries. Journal of Materials Chemistry A.

[57] Zhang Y., Ma X., Wen P., Liu Y., Lin X. (2025). A Dual Fluoropolymer
Composites as Organic Artificial Solid-Electrolyte Interphase for
Fast-Charging Lithium Metal Batteries. Energy
Fuels.

[58] Cui Y., Li Y., Zhou Y., Liu X., Lv J. (2025). Ion-Conducting and
Stretchable Organogel Polymer Interface Layer for Stabilizing Lithium
Metal Anodes via In Situ Polymerization Strategy. ACS Appl. Mater. Interfaces.

[59] Wang M.-x., Deng L.-j., Wang K.-l., Zou K., Yang T.-h., Liao X., Xiao R.-g., Ke X. (2026). Design and
construction
of organic-inorganic composite artificial solid electrolyte interface
films with high ionic conductivity for lithium-metal batteries. Electrochim. Acta.

[60] Pulkkinen P. M. S., Wiktorowicz S., Aseyev V., Tenhu H. (2013). Complexation of calix[4]­arene
protected gold nanoparticles with pyridinium and bipyridinium compounds. RSC Adv..

[61] Chen H., Liu P., Li H., Zhang H., Daniel S., Zeng Z. (2013). Fluorocarbon
and Hydrocarbon N-Heterocyclic (C5-C7) Difluorooxymethylene-Bridged
Liquid Crystals. Eur. J. Org. Chem..

