# Supplementary material for: Chain Conformation and Crosslink Density as Independent Mechanistic Levers in Comb-Brush Particle Artificial Solid Electrolyte Interfaces for Lithium–Metal Batteries
Source: ACS Appl Polym Mater. 2026 Aug 19;8(16):14061–72. doi: 10.1021/acsapm.6c02177 (PMC13532192; doi:10.1021/acsapm.6c02177)
Supplement: Supplementary file 1 [file ap6c02177_si_001.pdf]

Supporting Information

Chain Conformation and Crosslink-Density as Independent Mechanistic Levers in Comb-Brush Particle Artificial Solid Electrolyte Interfaces for Lithium-Metal Batteries

Verena Kempkes[a], Hanshu Wu[a], Jirameth Tarnsangpradit[b], Elizabeth E. DiFiglia[a], Bailey Williams[a], Kwangmo Go[a], Michael Bockstaller[b], Jay F. Whitacre*[b], Krzysztof Matyjaszewski*[a]

[a]Department of Chemistry
Carnegie Mellon University
4400 Fifth Avenue, Pittsburgh, PA 15213, USA
E-mail: matyjaszewski@cmu.edu

[b]Department of Materials Science and Engineering
Carnegie Mellon University
5000 Forbes Avenue, Pittsburgh, PA 15213, USA
E-mail: whitacre@andrew.cmu.edu

Table S1: Molecular properties of additional SiO_2-*g*-(DMAEMA-*co*-$OEOMA_{500}$) samples for scaling factor determination.

**Entry**	**$M_{n,app}$[a] [kDa]**	**Đ [ ]**	**f_{inorg}[b] [%]**	**GD[c] [nm^{-2}]**	**Brush height [nm]**
N_0[1]	52.2	1.42	8.42	0.72	14.05 ± 2.76
N_{0-1}[1]	95.9	1.39	7.82	0.43	25.49 ± 7.52
N_{0-2}[1]	111.4	1.58	4.23	0.71	32.52 ± 6.06
N_{20}	65.3	1.40	7.23	0.69	18.18 ± 4.55
N_{20-1}	44.1	1.46	8.99	0.80	13.75 ± 5.12
N_{20-2}	59.3	1.23	7.12	0.77	17.49 ± 2.65
N_{25}	72.6	1.45	5.88	0.76	14.77 ± 2.50
N_{25-1}	58 9	1.63	7.49	0.73	13.26 ± 5.29
N_{25-2}	84.5	1.46	7.17	0.54	17.09 ± 5.22
N_{30}	54.8	1.35	6.31	0.95	13.11 ± 2.53
N_{30-1}	35.4	1.54	12.08	0.71	12.62 ± 4.91
N_{30-2}	76.7	1.47	6.78	0.63	18.46 ± 5.00
N_{35}	59.2	1.36	6.47	0.85	16.38 ± 5.12
N_{35-1}	43.7	1.49	10.85	0.66	12.30 ± 5.55
N_{35-2}	78.2	1.54	6.85	0.61	16.73 ± 2.82

[a]Determined via gel permeation chromatography in DMF at 50°C, calibrated using polymethylmethacrylate standards. [b]Determined via thermogravimetric analysis. [c]Calculated by equation 1.

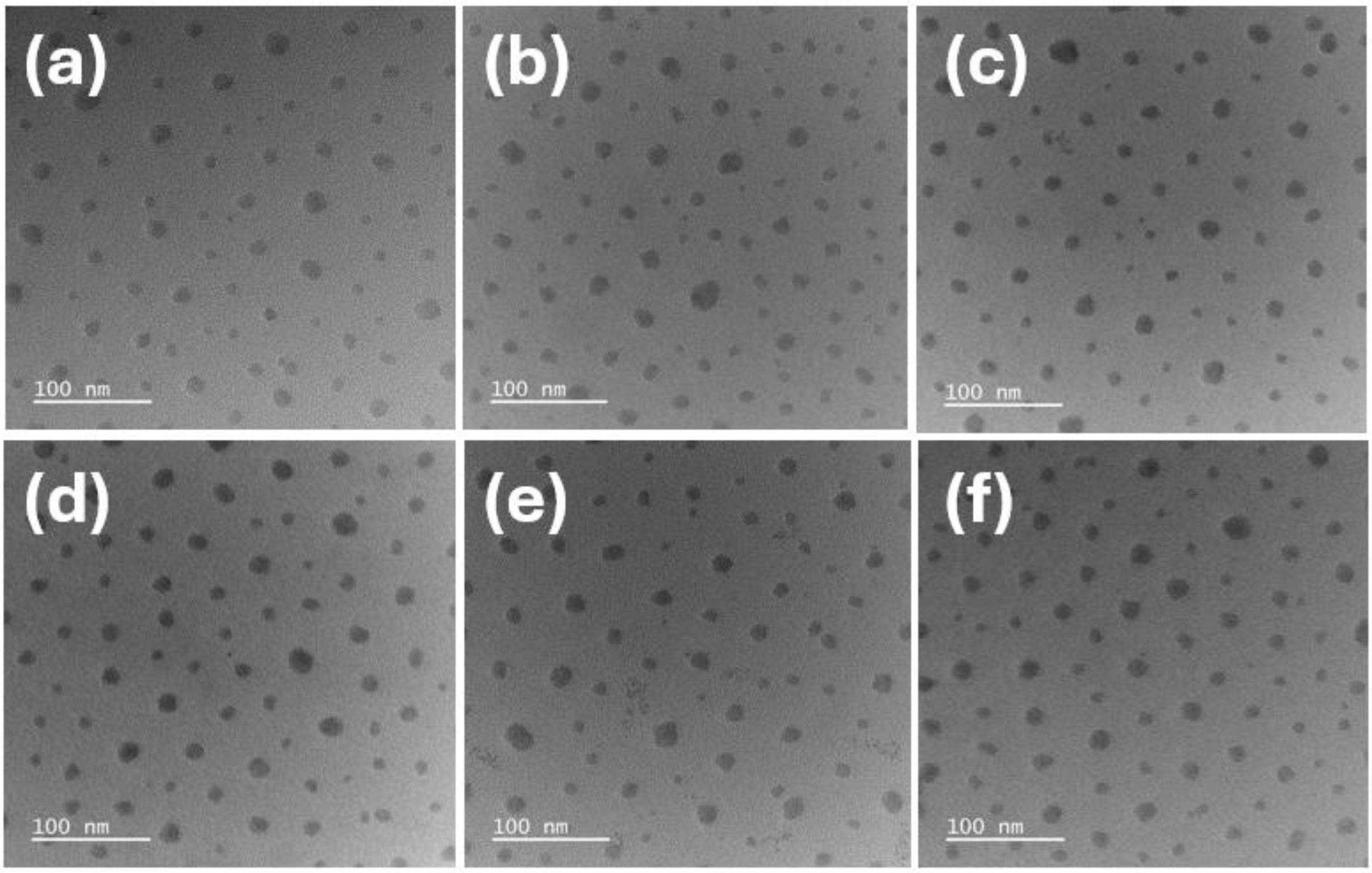

Fig. S1: TEM images of N_5 (a), N_{10} (b), N_{20} (c), N_{25} (d), N_{30} (e) and N_{35} (f) after casting on a carbon-coated copper grad from a THF solution and annealing under vacuum at 120°C overnight.

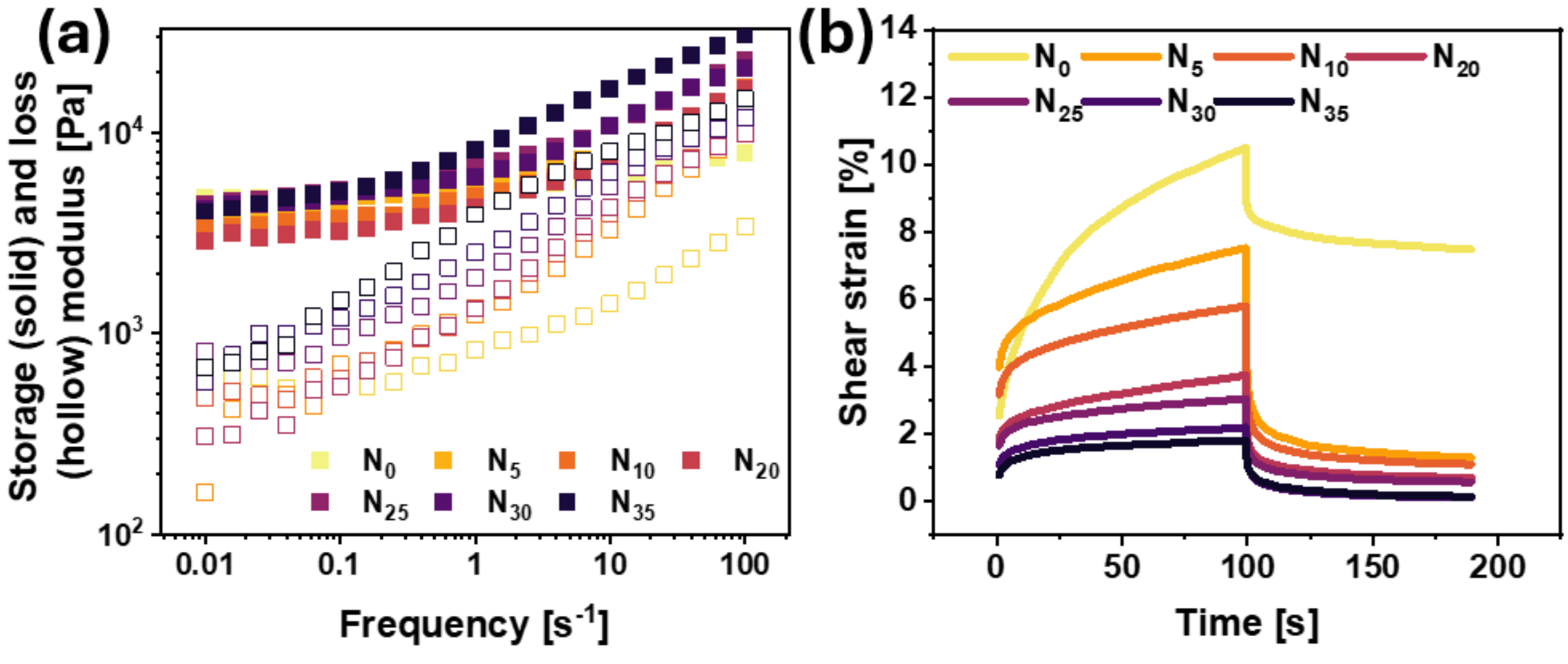

Fig. S2: Frequency sweeps from 0.01 to 100 s^{-1} of CBP materials at room temperature (a) and creep tests of CBP materials at room temperature by applying a shear stress of 100 Pa for 100 s and 0 Pa for 75 s (b).

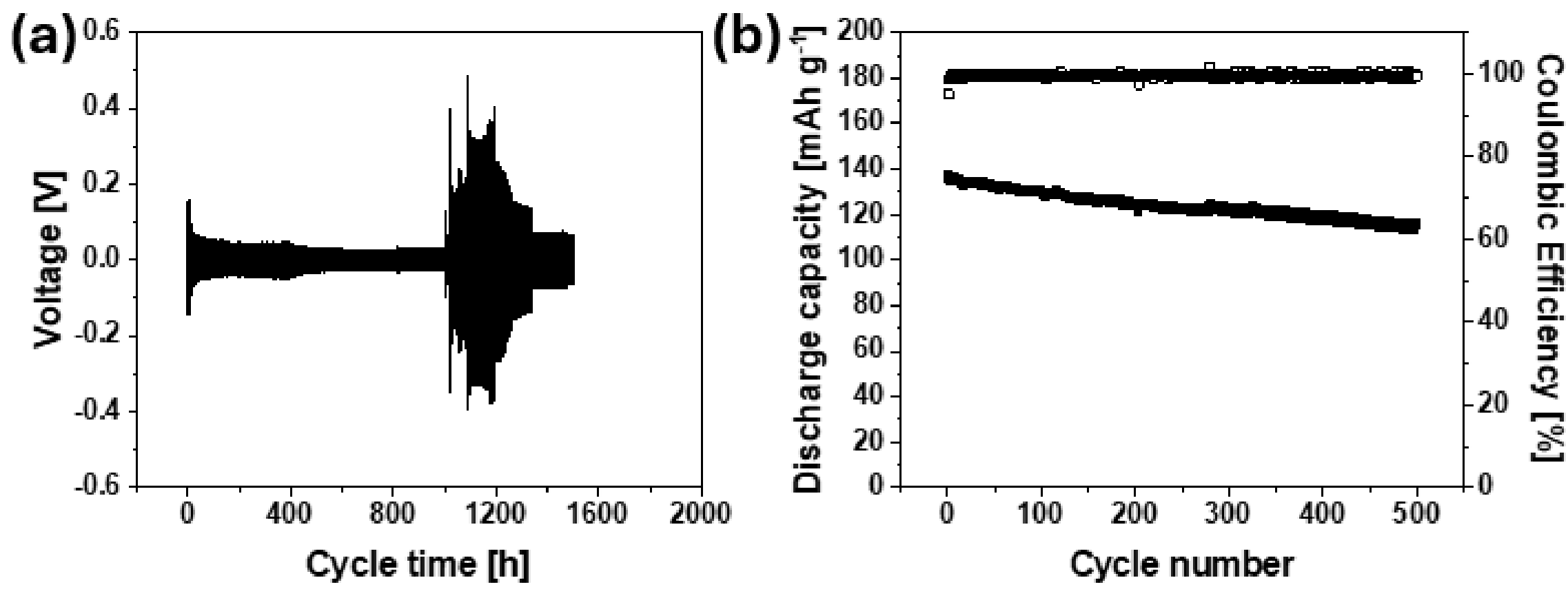

Fig. S3: Symmetric cycling of Li|Li cell at 1 mA cm^{-2} and 0.5 mAh cm^{-2} (a) and cycling stability (discharge capacity with filled squares and Coulombic efficiency with hollow squares) of Li|LFP cells at 0.5C (b).

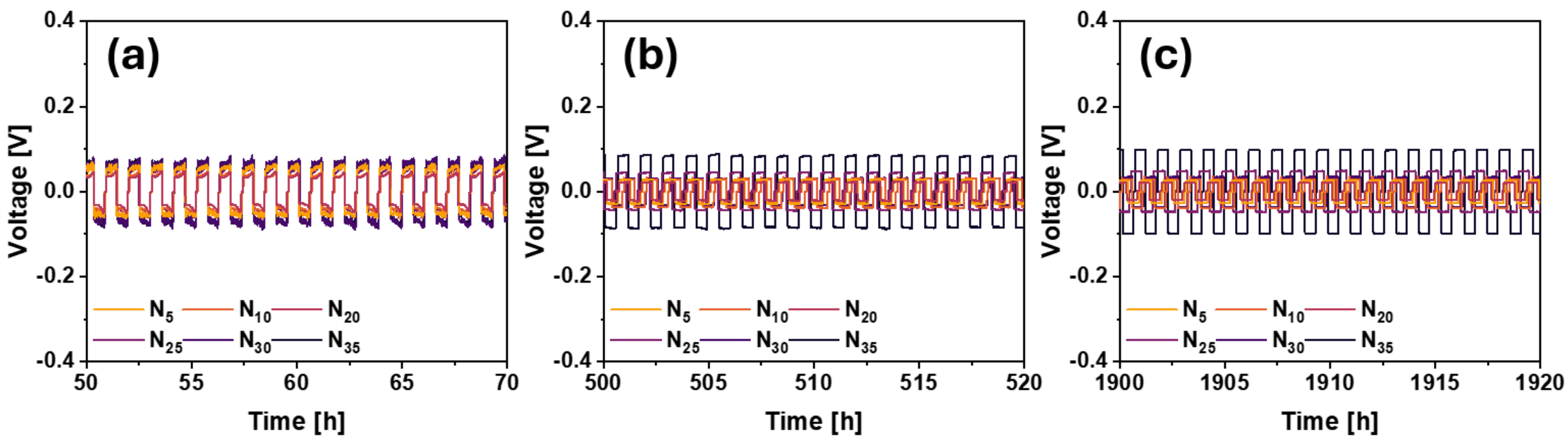

Fig. S4: Magnified curves for cycling in symmetric cycling in CBP@Li|CBP@Li cells of 50-70 h (a), 500-520 h (b) and 1900-1920 h (c).

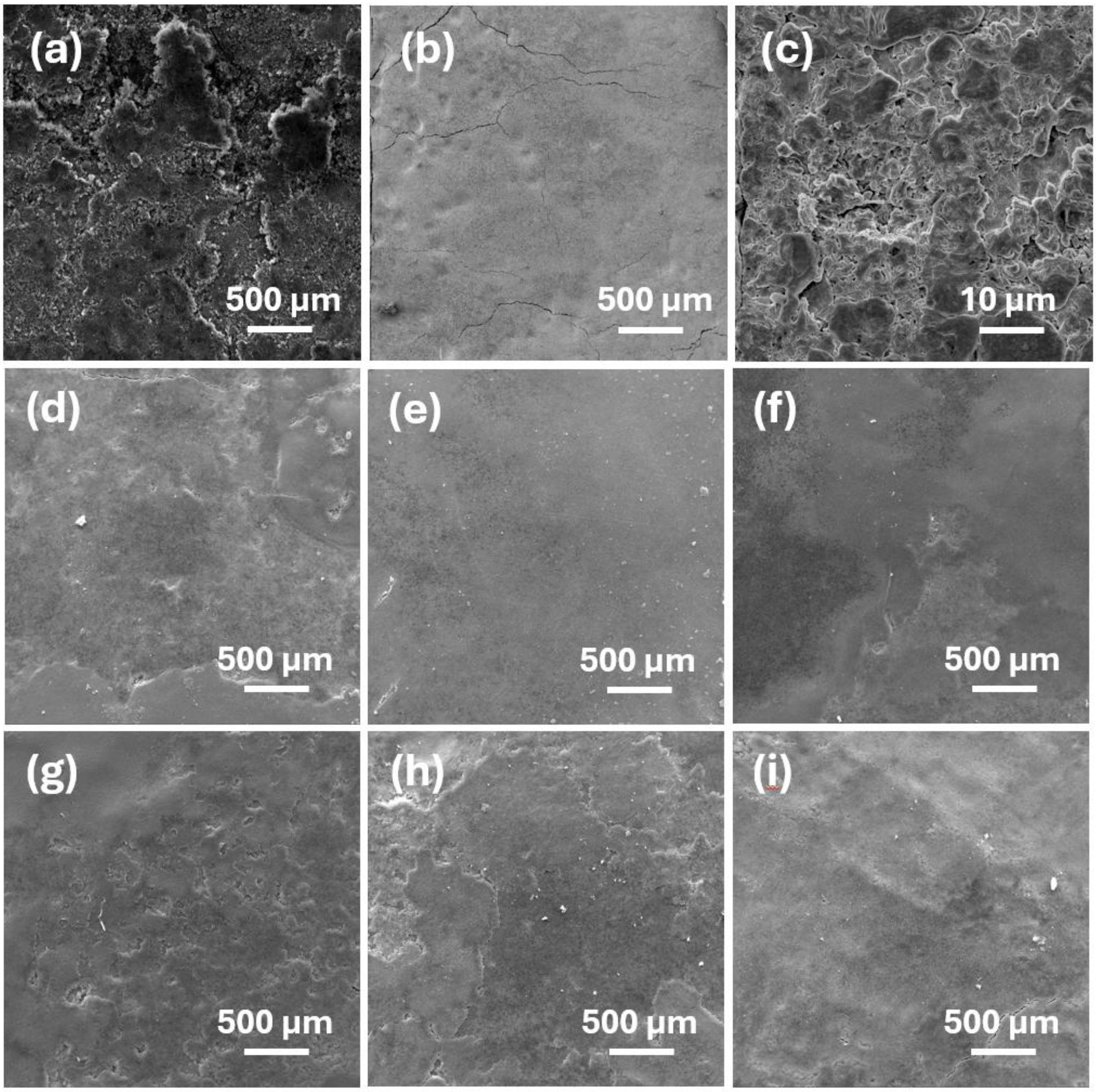

Fig. S5: SEM images of lithium anode surfaces after 2000 h of cycling for bare lithium (a) and protected by N_0 (b+c), N_5 (d), N_{10} (e), N_{20} (f), N_{25} (g), N_{30} (h) and N_{35} (i).

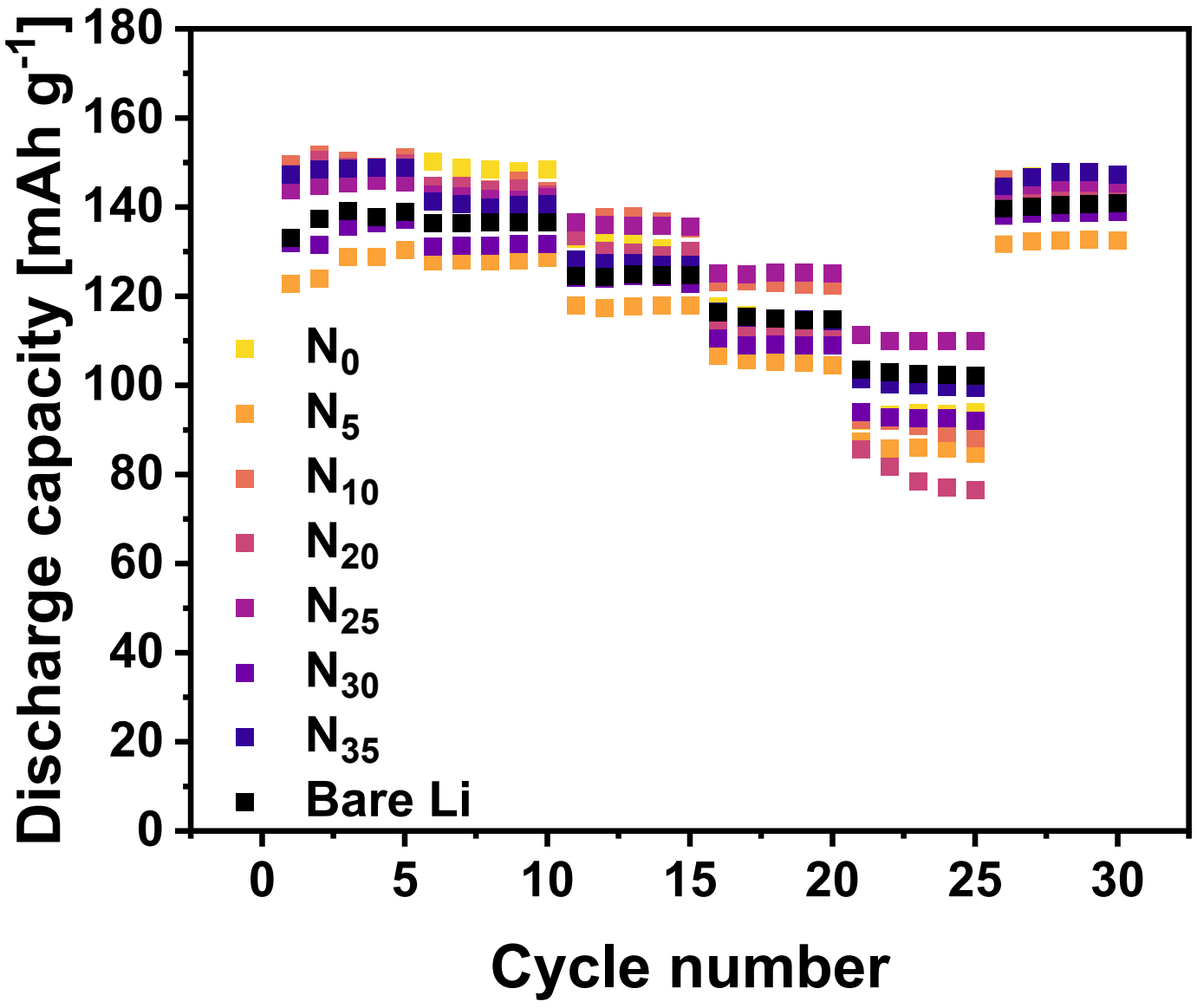

Fig. S6: Rate capability test of Li|LFP and CBP@Li|LFP cells at C-rates of 0.1C, 0.2C, 0.5C, 1C and 2C.

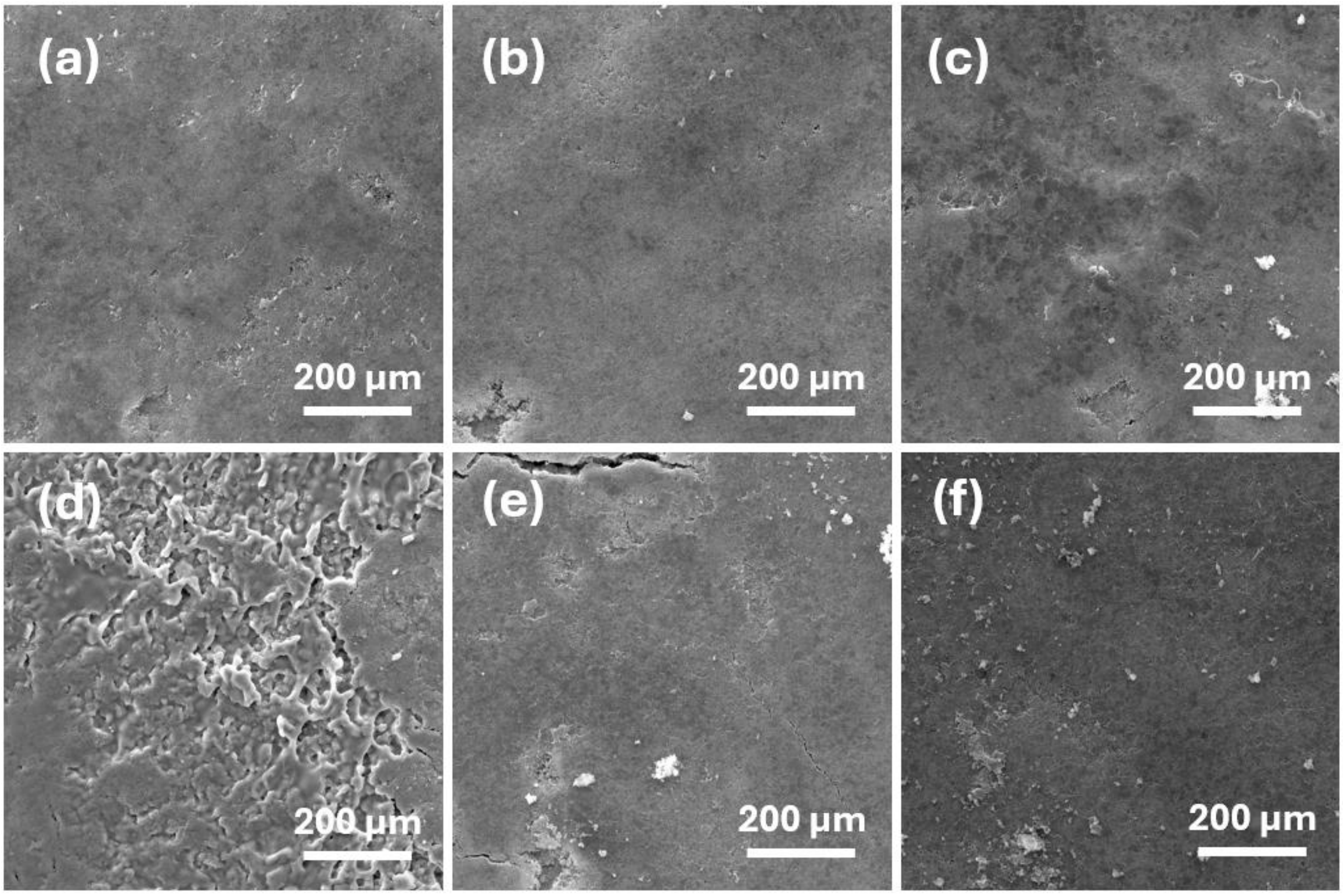

Fig. S7: SEM images of lithium anode surfaces after 2000 h of cycling protected by N_5 (a), N_{10} (b) and N_{20} (c) crosslinked by 1,6-dibromohexane and N_5 (d), N_{10} (e) and N_{20} (f) crosslinked by 1,4-butaneditriflate.

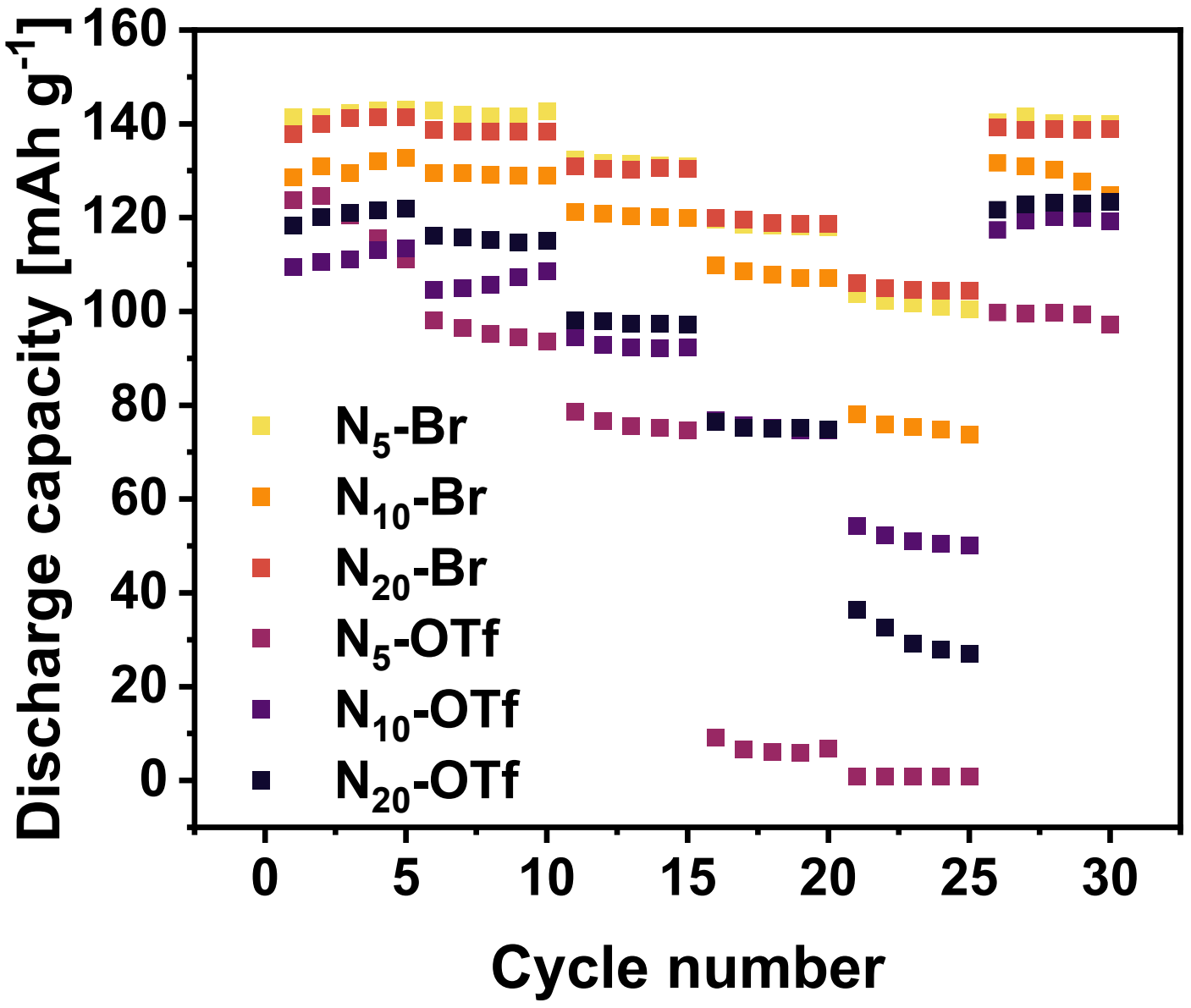

Fig. S8: Rate capability test of crCBP@Li|LFP cells at C-rates of 0.1C, 0.2C, 0.5C, 1C and 2C.

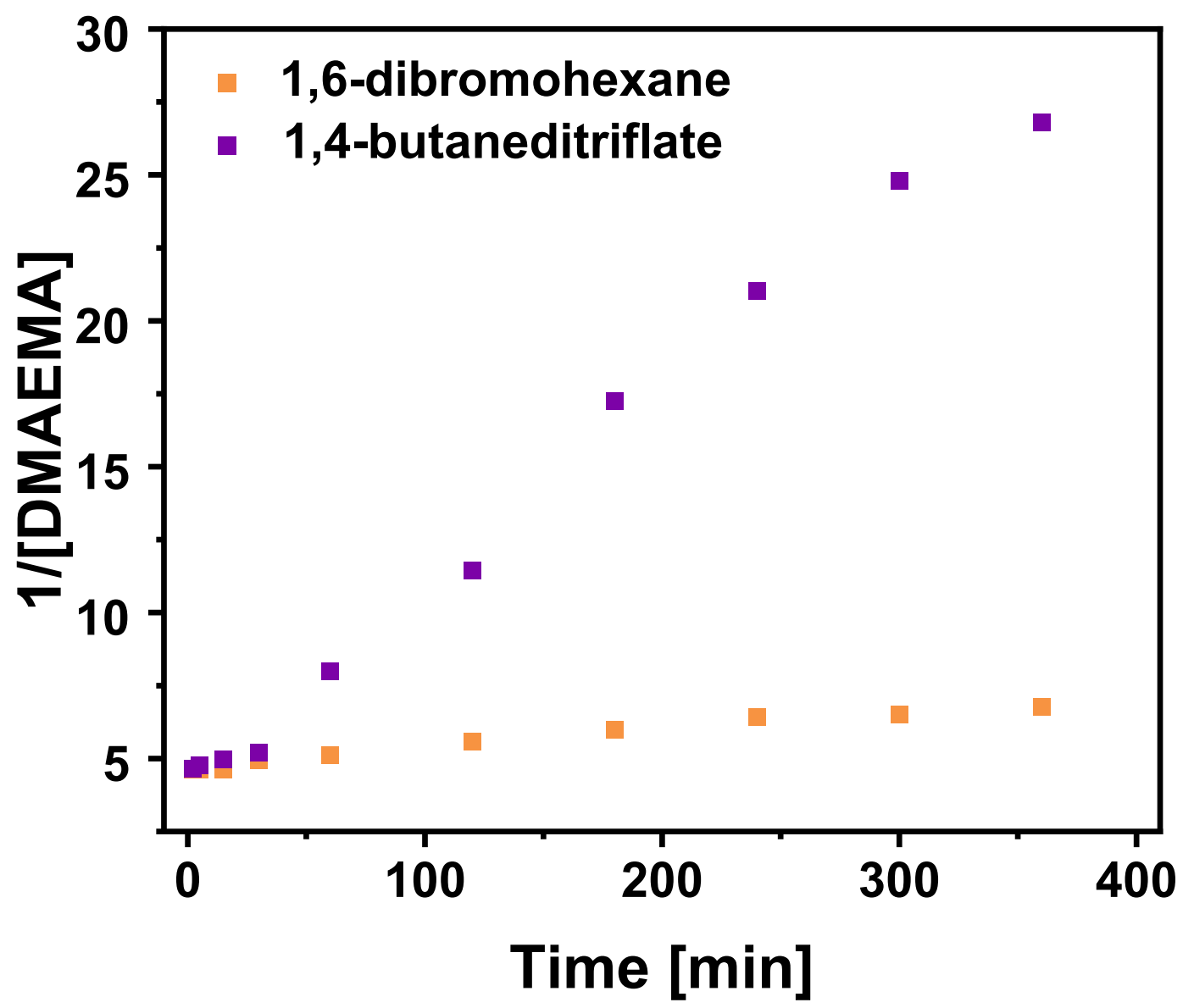

Fig. S9: Second order kinetic plots for the quaternization of DMAEMA in acetone-d6 (0.01M crosslinker, 0.02M DMAEMA).

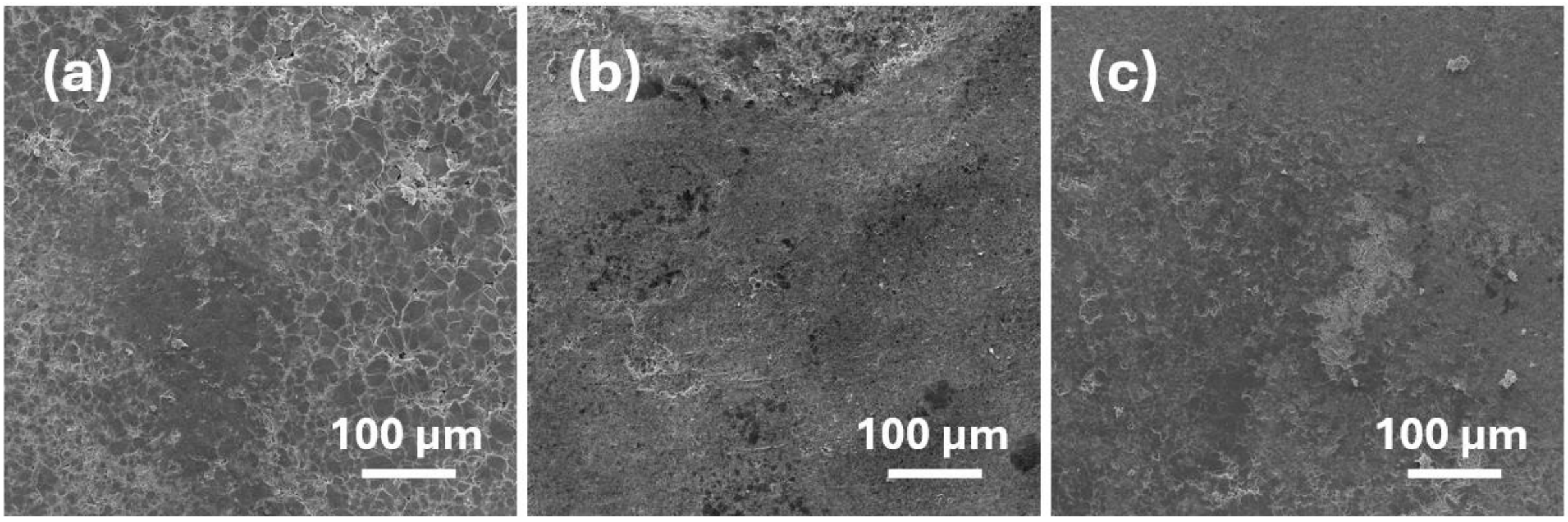

Fig. S10: SEM images of lithium anode surfaces after 1000 h of cycling protected by N_5 (a), N_{10} (b) and N_{20} (c) using Br-PEO_{2k}-Br as the crosslinker.

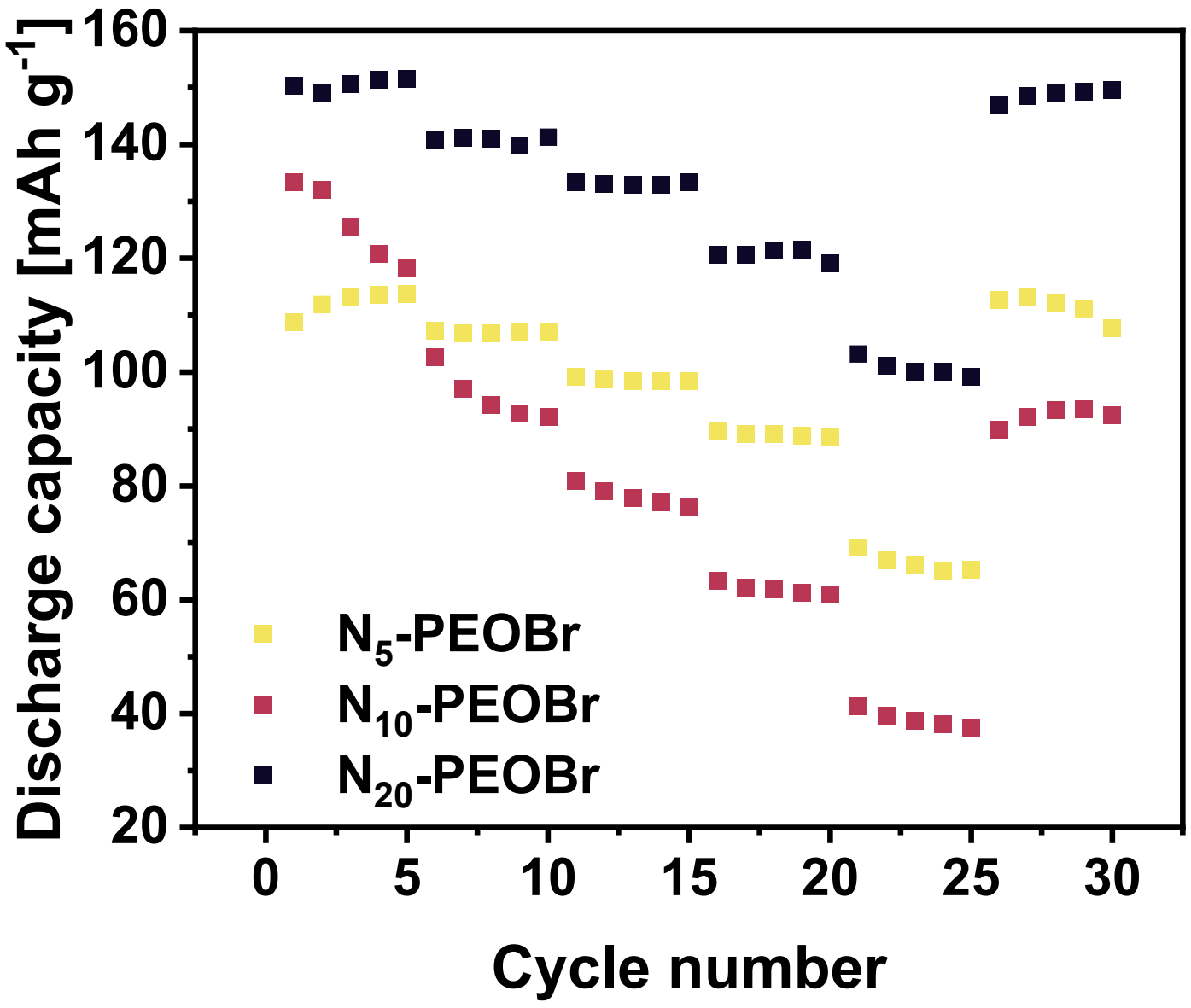

Fig. S11: Rate capability test of crCBP@Li|LFP cells with Br-PEO_{2k}-Br at C-rates of 0.1C, 0.2C, 0.5C, 1C and 2C.

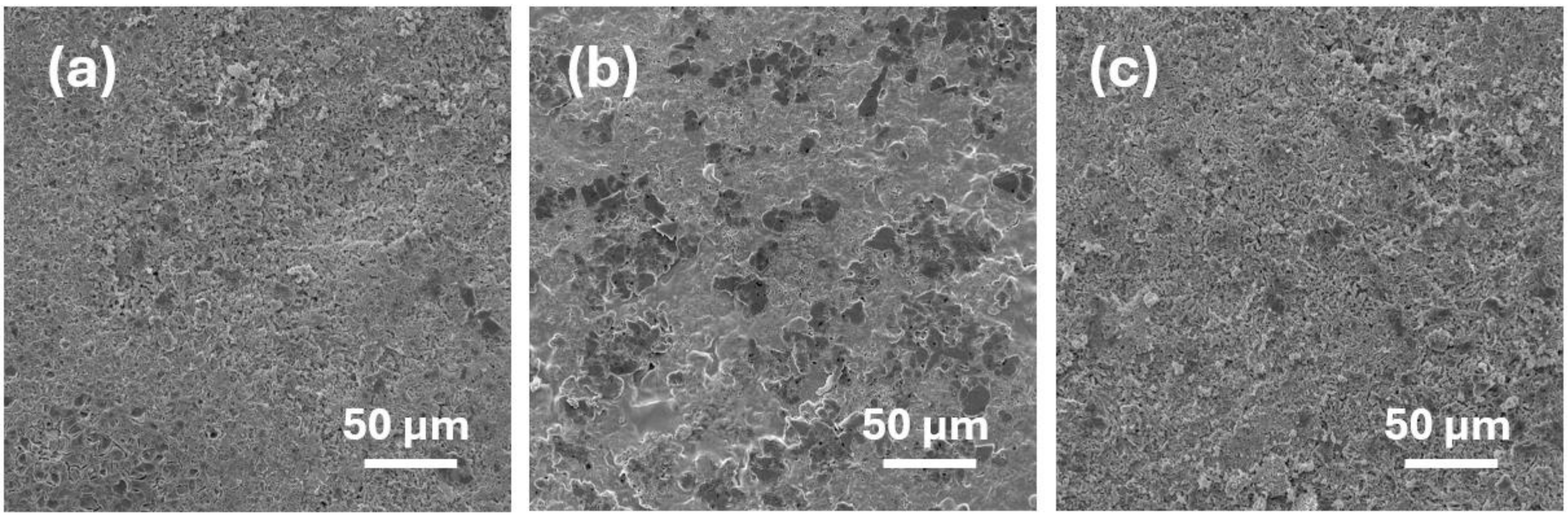

Fig. S12: SEM images of lithium anode surfaces after 100 cycles protected by N_5 (a), N_{10} (b) and N_{20} (c) using Br-PEO_{2k}-Br as the crosslinker.

Reference

1. Kempkes, V.; Liu, T.; Tarnsangpradit, J.; Li, S.; Bockstaller, M. R.; Whitacre, J. F.; Matyjaszewski, K., Effect of Bottlebrush Particle Architecture on Their Efficiency as Protective Layers in Li-Metal Batteries. *ACS Applied Polymer Materials* **2025,** *7* (20), 13885-13895.
